# KRAS allelic imbalance drives tumour initiation yet suppresses metastasis in colorectal cancer in vivo

**DOI:** 10.1038/s41467-023-44342-4

**Published:** 2024-01-02

**Authors:** Arafath K. Najumudeen, Sigrid K. Fey, Laura M. Millett, Catriona A. Ford, Kathryn Gilroy, Nuray Gunduz, Rachel A. Ridgway, Eve Anderson, Douglas Strathdee, William Clark, Colin Nixon, Jennifer P. Morton, Andrew D. Campbell, Owen J. Sansom

**Affiliations:** 1 Cancer Research UK Scotland Institute, Glasgow, UK; 2 Institute of Biotechnology, HiLIFE, University of Helsinki, Helsinki, Finland; 3 School of Cancer Sciences, University of Glasgow, Glasgow, UK

**Keywords:** Colon cancer, Cancer models, Oncogenes

## Abstract

Oncogenic *KRAS* mutations are well-described functionally and are known to drive tumorigenesis. Recent reports describe a significant prevalence of *KRAS* allelic imbalances or gene dosage changes in human cancers, including loss of the wild-type allele in *KRAS* mutant cancers. However, the role of wild-type KRAS in tumorigenesis and therapeutic response remains elusive. We report an in vivo murine model of colorectal cancer featuring deletion of wild-type *Kras* in the context of oncogenic *Kras*. Deletion of wild-type *Kras* exacerbates oncogenic KRAS signalling through MAPK and thus drives tumour initiation. Absence of wild-type *Kras* potentiates the oncogenic effect of KRASG12D, while incidentally inducing sensitivity to inhibition of MEK1/2. Importantly, loss of the wild-type allele in aggressive models of KRASG12D-driven CRC significantly alters tumour progression, and suppresses metastasis through modulation of the immune microenvironment. This study highlights the critical role for wild-type *Kras* upon tumour initiation, progression and therapeutic response in *Kras* mutant CRC.

## Abstract (web summary)

The function of wild-type KRAS in KRAS mutant cancers remains to be explored. Here, the authors show that deletion of the tumour-suppressive wild-type *Kras* in a KRASG12D driven colon cancer model exacerbates tumour initiation in a MAPK dependent manner, while acting to suppress metastasis through impaired immune suppression.

## Introduction

It is known that oncogene allelic imbalance is a frequent event in cancer cells, known to impact key oncogene loci such as KRAS and BRAF^1^. These allelic imbalances can result from genomic gains or losses, focal amplifications at the oncogene loci or loss of heterozygosity impacting the wild-type allele. Nonetheless, the functional and therapeutic consequences of such imbalances are poorly understood. Mutations in KRAS oncogene are frequently detected in many cancers, including colorectal adenocarcinoma (CRC) and pancreatic ductal adenocarcinoma (PDAC). Hotspot mutations involving codons 12, 13, 61 and 146 of KRAS lead to constitutive activation of KRAS and hyperactivation of many downstream effector signalling pathways, including MAPK and PI3K-AKT pathways^2,3^. Biochemically, these hotspot mutations have been shown to increase the abundance of GTP-bound active KRAS, through altered GTP hydrolysis and nucleotide exchange rates based on the type of mutation^4^. Recent reports have shown that as a likely consequence of their distinct biochemical properties, each specific KRAS mutation has a unique transforming potential and tumour-promoting capacity dependent on the tissue or cancer type^5–7^.

It is now well established that the frequency of KRAS mutation differs widely based on the tissue of origin (40% of CRC and 93% of pancreatic cancers)^8^. We and others have previously shown that oncogenic *Kras* cooperates with *Apc* loss in driving colorectal tumorigenesis^9–11^. The majority of studies to date have focused on gain-of-function effects of *KRAS* mutations^12^. Unfortunately, and despite the recent successes with KRASG12C isoform-specific inhibitors, it is widely accepted that mutant KRAS is strongly associated with resistance to therapies, particularly those targeting upstream or downstream signalling nodes such as EGFR, MEK, PI3K and mTOR^13–15^. This said, given their position at the nexus of many key oncogenic and growth-promoting signalling pathways, significant efforts are underway both to directly target mutant RAS oncoproteins, and to target signalling through the wild-type molecule^16,17^.

Recent work from several labs has highlighted the heterogeneous properties of KRAS alterations in human cancer, having successfully modelled a spectrum of KRAS mutations in relevant model systems^5–7^. In addition to this spectrum of oncogenic KRAS mutations, evaluation of specific pro- or anti-tumourigenic roles for the wild-type RAS proteins in the context of oncogenic RAS across cancer types has been controversial. It has previously been proposed that wild-type KRAS can exhibit tumour-suppressive characteristics in cancer^18^. More recently, it was also shown that KRAS dimerization is required for the function of oncogenic KRAS^19^. In addition, it has become clear that copy number alterations at *KRAS* such as copy number gain at the mutant allele, loss of heterozygosity, or broader allelic imbalance can result in enhanced fitness of cancer cell lines^1,20–22^. Indeed, it has been demonstrated that *KRAS* gene dosage can determine phenotypic characteristics and influence outcome of pancreatic cancer models in vivo^23,24^. Nonetheless, a clear understanding of the mechanistic basis of wild-type KRAS function in the processes of tumour initiation and progression of KRAS mutant tumours is yet to be defined.

Understanding the function of wild-type KRAS is essential to identify effective therapeutic strategies for oncogenic *KRAS*-driven cancers and potential mechanisms of resistance to KRAS targeting therapies. Here, we demonstrate a critical role for wild-type KRAS in tumour initiation and progression of mutant KRAS-driven tumours. Using genetically engineered mouse models (GEMMs), we show that in the presence of an oncogenic *Kras* allele loss of the wild-type *Kras* allele augments oncogenic *Kras* signalling, leading to increased tumour initiation in vivo. Furthermore, utilising KRAS-driven colorectal cancer models we show that loss of wild-type KRAS promotes sensitivity to MEK inhibition in vivo. Finally, we show that deletion of wild-type *Kras* allele significantly alters the progression of advanced late-stage KRAS-driven colorectal tumours. Collectively, our studies provide important insights into KRAS biology and reveal a critical role for wild-type KRAS in the therapeutic resistance of KRAS-driven cancers.

## Results

### Allelic balance of mutant and wild-type KRAS affects homoeostasis in the murine intestine

**Fig. 1:**
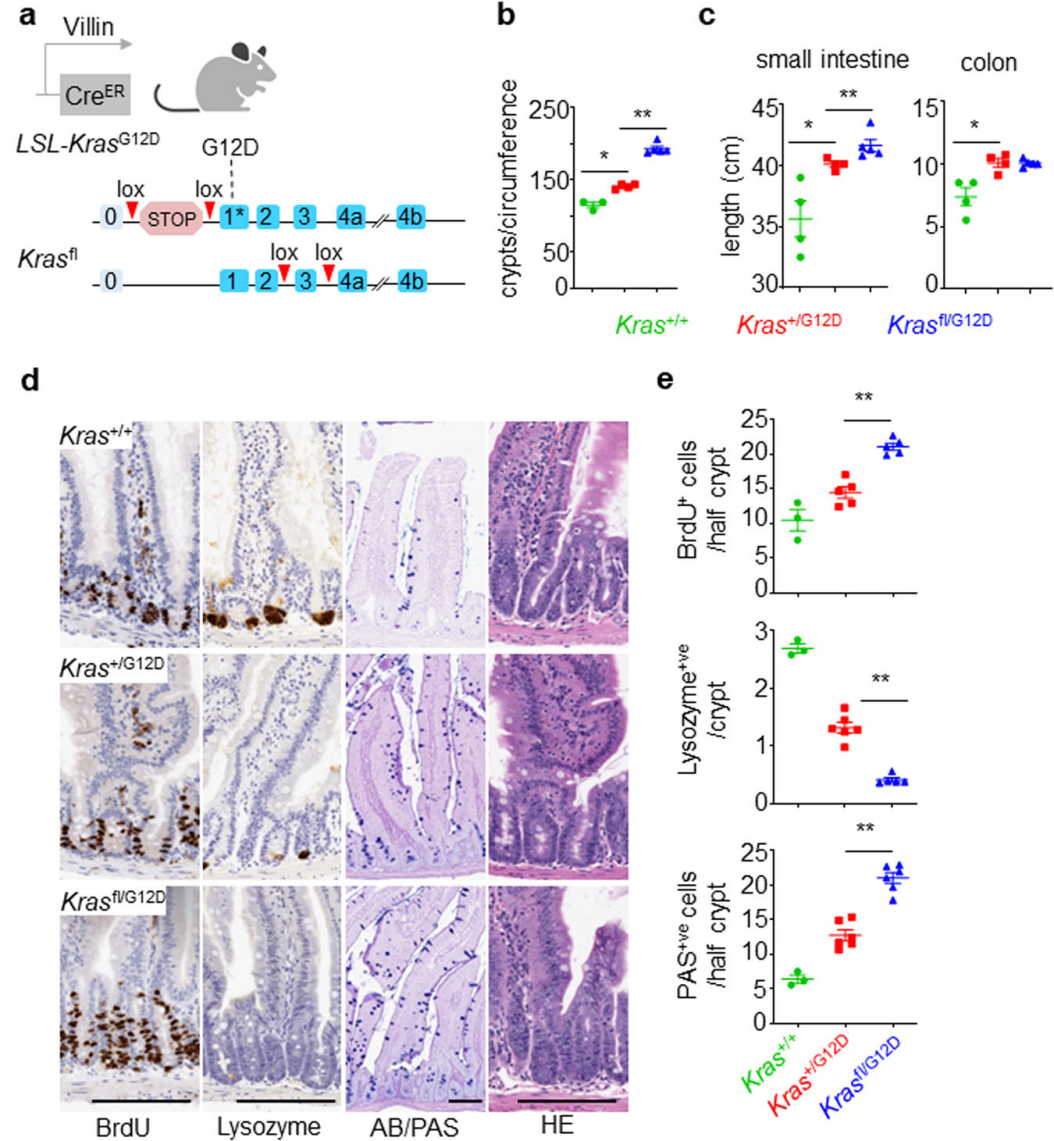
Wild-type *Kras* deletion alters *Kras*^G12D^ mutant intestinal homoeostasis. **a** Schematic representing the generation of *Kras*^fl/G12D^ mice: Villin Cre, Cre recombinase; ER oestrogen receptor; lox, Cre-Lox recombination site; LSL Lox-Stop-Lox cassette. Created with BioRender.com. **b** Number of crypts per circumference from at least 25 of the small intestine of *Kras*^+/+^, *Kras*^+/G12D^ and *Kras*^fl/G12D^ mice, sampled 30 days post Cre-induction (*Kras*^+/+^, *n* = 3, 2M,1F; *Kras*^+/G12D^, *n* = 4, 2M, 2F; and *Kras*^fl/G12D^, *n* = 5, 4M, 1F). Data are ± s.e.m. *P = 0.0286, **P = 0.0079, one-way Mann–Whitney *U* test. Experiments carried out on C57BL/6J background ≥ N2. **c** Length (cm) of small intestine (SI) and colon of *Kras*^+/+^, *Kras*^+/G12D^ and *Kras*^fl/G12D^ mice, sampled 30 days post Cre-induction (*Kras*^+/+^, *n* = 4, 3M, 1F; *Kras*^+/G12D^, *n* = 4, 2M, 2F; and *Kras*^fl/G12D^, *n* = 5, 3M, 2F). Data are mean ± s.e.m. *P = 0.0143, **P = 0.0079 one-way Mann–Whitney *U* test. Experiments carried out on C57BL/6J background ≥ N2. **d** Representative 5-bromo-2′-deoxyuridine (BrdU), Lysozyme, Alcian blue/periodic acid-Schiff (AB/PAS) and H&E staining of *Kras*^+/+^, *Kras*^+/G12D^ and *Kras*^fl/G12D^ mouse small intestine, sampled 30 days post Cre-induction. Scale, 100 μm. **e** Top: Number of BrdU-positive cells from at least 25 half-crypts in SI from (**d**). Data are mean ± s.e.m, (*Kras*^+/+^, *n* = 3, 2M, 1F; *Kras*^+/G12D^, *n* = 5, 3M, 2F; *Kras*^fl/G12D^ *n* = 5, 3M, 2F), **P = 0.004, one-way Mann–Whitney U test. Middle: Number of Lysozyme-positive cells from at least 25 crypts in SI of *Kras*^+/+^, *Kras*^+/G12D^ and *Kras*^fl/G12D^ in SI from (**d**) Data are mean ± s.e.m, (*Kras*^+/+^, *n* = 3, 2M, 1F; *Kras*^+/G12D^, *n* = 6, 3M, 3F; *Kras*^fl/G12D^ *n* = 5, 3M, 2F), **P = 0.0022, one-way Mann–Whitney U test. Bottom: Number of PAS-positive cells from at least 25 half-crypts in SI. *Kras*^+/+^, *Kras*^+/G12D^ and *Kras*^fl/G12D^ in SI from (**d**) Data are mean ± s.e.m, (*Kras*^+/+^, *n* = 3, 2M, 1F; *Kras*^+/G12D^, *n* = 6, 4M, 2F; *Kras*^fl/G12D^ *n* = 6, 4M, 2F). **P = 0.0011, one-way Mann–Whitney *U* test. Experiments carried out on C57BL/6J background ≥ N2. Source data are provided as a Source Data file.

To accurately model the contribution of wild-type KRAS in CRC, we designed a GEMM that allows selective deletion of the wild-type *Kras* while expressing an oncogenic *Kras*^LSL-G12D^ allele (hereafter *Kras*^G12D^). Here, the wild-type *Kras* allele is replaced by a conditional *Kras*^flox^ allele to generate *Kras*^fl/LSL-G12D^ (hereafter referred to as *Kras*^fl/G12D^) (Fig. 1a). Recombination of these alleles is targeted to the intestinal epithelium through activity of a tamoxifen-inducible Cre recombinase, expressed under the control of the *Villin* promoter (villin-creERT2) (Fig. 1a). Using this model we confirmed the impact of the oncogenic *Kras*^G12D^ mutation upon intestinal epithelial homoeostasis, and subsequently went on to characterise any modification of this phenotype elicited by *Kras*^fl/G12D^. Intestinal tissue was sampled from mice at 30 days post-induction, with mutant KRAS found to promote enterocyte proliferation and robustly suppress Paneth cell differentiation in the small intestine as a consequence of increased MAPK signalling, consistent with previous reports^25^ (Fig. 1b–e). These features are further exacerbated by deletion of the wild-type copy in *Kras*^fl/G12D^ mice, exemplified by increased proliferation in the intestinal crypt (BrdU^+^) (Fig. 1d, e). Moreover, Lysozyme and periodic acid-Schiff (PAS) or Alcian blue (AB) stains indicated further suppression of the Paneth cell lineage and increased abundance of secretory goblet cells (Fig. 1d, e). These in vivo data are suggestive of a role for wild-type KRAS in restraining the impact of oncogenic *KRAS* mutation on the intestinal epithelium, which in turn translates into quantitative phenotypic differences.

Allelic imbalance is reported to be a common feature associated with many oncogenes in addition to *KRAS*^1^. For example, BRAF allelic imbalance is reported to occur in 40% of BRAF mutant skin cancers^26^. Therefore, we tested whether *Braf* allelic imbalance could also impact intestinal homoeostasis. Here, we assessed the impact of a conditional oncogenic *Braf*^V600E^ allele, alone or in combination with a conditional *Braf* targeting allele (henceforth referred to as *Braf*^*fl/V600E*^), or when bred to homozygosity (*Braf*^V600E/V600E^), again under the control of villin-creERT2. To assess the impact of *Braf*^V600E^ gene dosage, intestinal tissues were sampled from *Braf*^+/V600E^, *Braf*^fl/V600E^, *Braf*^V600E/V600E^ or control mice at a time point 3 days post-induction recombination. As previously reported, we found that *Braf*^V600E/+^ promotes proliferation in the intestinal crypt (BrdU^+^), indeed to a greater degree than *Kras*^G12D/+^ at this time point (Supplementary Fig. 1a, b)^27,28^. In line with the phenotypes observed in KRAS mutant intestine, we find that altering allelic balance in favour of oncogenic *Braf*, either through breeding to homozygosity (*Braf*^V600E/V600E^), or through conditional deletion of the wild-type allele (*Braf*^fl/V600E^) leads to significant increase in crypt cell proliferation and loss of Lysozyme-positive Paneth cells (Supplementary Fig. 1a, b). However, BRAF activation does not significantly alter the abundance of secretory goblet cells in the intestinal epithelium (Supplementary Fig. 1a, b). Collectively, these data show that allelic imbalance at oncogene loci akin to that seen in human cancer exacerbates specific oncogene-associated and cancer-related phenotypes.

### Wild-type Kras deletion in the presence of oncogenic KRASG12D and Wnt activation accelerates tumorigenesis via MAPK signalling

**Fig. 2:**
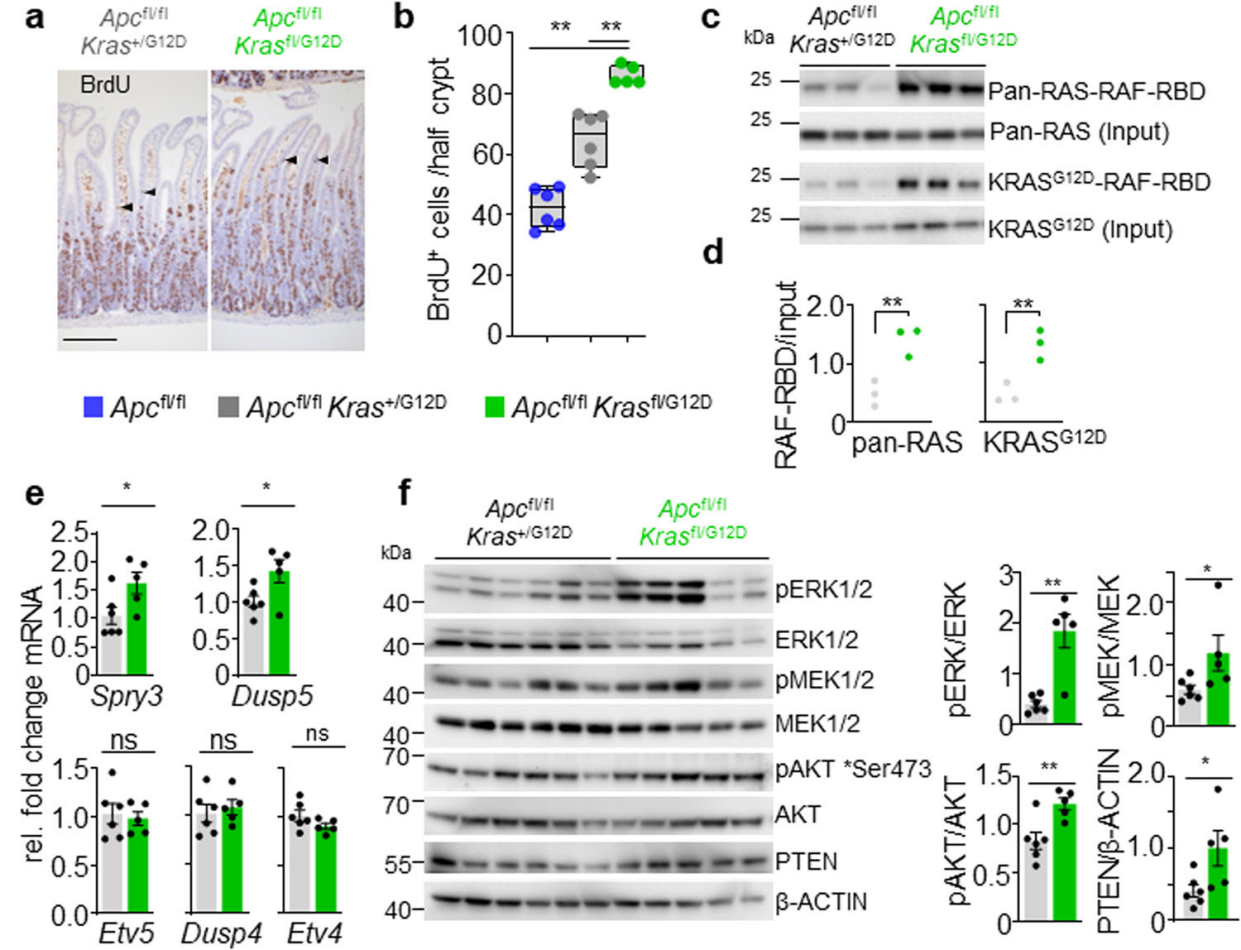
Wild-type *Kras* deletion increases proliferation, active KRAS and MAPK signalling. **a** Representative H&E and BrdU IHC of *Apc*^fl/fl^ *Kras*^+/G12D^ and *Apc*^fl/fl^ *Kras*^fl/G12D^ mice sampled 3 days post Cre-induction. Arrowheads indicate de-differentiating cells in the villi. Scale, 100 μm. **b** Boxplots showing BrdU-positive cells from at least 25 half-crypts in SI in *Apc*^fl/fl^*, Apc*^fl/fl^ *Kras*^+/G12D^ and *Apc*^fl/fl^ *Kras*^fl/G12D^ mice. Boxes depict interquartile range, central line indicates median and whiskers indicate minimum/maximum values *(Apc*^fl/fl^, *n* *=* 6, 4M, 2F*; Apc*^fl/fl^ *Kras*^+/G12D^, *n* = 6, 2M, 4F; and *Apc*^fl/fl^ *Kras*^fl/G12D^, *n* = 5, 1M, 4F mice). **P = 0.0022, **P = 0.0043. **c** RAF-Ras Binding Domain (RBD) agarose affinity purification assay of three biologically independent samples per condition from *Apc*^fl/fl^ *Kras*^+/G12D^ *and Apc*^fl/fl^ *Kras*^fl/G12D^ intestinal organoids. Pulldown of RAS-GTP with RAF-RBD agarose beads. Top: Precipitates were immunoblotted using a pan-RAS antibody and input pan-RAS served as loading control. RAS-GTP activation levels were quantified and normalised to pan-Ras loading control. *Apc*^fl/fl^ *Kras*^+/G12D^, *n* = 3, 2M, 1F; and *Apc*^fl/fl^ *Kras*^fl/G12D^, *n* = 3, 1M, 2F. Bottom: Precipitates were immunoblotted using a KRASG12D antibody and input KRASG12D served as loading control. KRASG12D-RAF-RBD levels were quantified and normalised to KRASG12D loading control. *Apc*^fl/fl^ *Kras*^+/G12D^, *n* = 3, 3M; and *Apc*^fl/fl^ *Kras*^fl/G12D^, *n* = 3, 1M, 2F. **d** Quantification of RAF-RBD assay (left) and KRASG12D immunoblot (right from **c**), **P = 0.0045 (pan-RAS), **P = 0.0044 (KRASG12D). **e** qRT-PCR analysis of *Etv5, Etv4, Dusp4, Dusp5* and *Spry3* in *Apc*^fl/fl^ *Kras*^+/G12D^ (*n* = 6, 3M, 3F) and *Apc*^fl/fl^ *Kras*^fl/G12D^ (*n* = 5, 1M, 4F) intestinal organoids. Data are mean ± s.e.m. Transcript levels were normalised to *Gapdh*. *P = 0.0260 (*Spry3*) *P = 0.0411 (*Dusp5*). **f** Left: Immunoblots showing PTEN, pAKT (Ser473), AKT, pERK1/2, ERK1/2, pMEK1/2, MEK1/2 and ß-actin in *Apc*^fl/fl^ *Kras*^+/G12D^ *and Apc*^fl/fl^ *Kras*^fl/G12D^ intestinal organoids. 6 *Apc*^fl/fl^ *Kras*^+/G12D^ *and* 5 *Apc*^fl/fl^ *Kras*^fl/G12D^ biological replicates per group, each lane represents organoids generated from individual mice from genotype indicated. Right: Quantification of immunoblots, phosphorylated proteins were normalised to total protein levels. Data are mean ± s.e.m. **P = 0.0043 (pERK/ERK), *P = 0.0152 (pMEK/MEK), **P = 0.0087 (pAKT/AKT), *P = 0.015 (PTEN/ß-actin). **b**, **d**, **e**, **f** using one-way Mann–Whitney *U* test. Source data are provided as a Source Data file.

Given the observed impact upon normal intestinal homoeostasis driven by altered gene dosage at oncogenic loci, we investigated whether these events might cooperate with the concomitant loss of the tumour suppressor gene *Apc*. The *Kras*^fl/G12D^ mouse line described above was bred to the well-characterised mouse line bearing conditional deletion of the tumour suppressor gene *Apc* (villin-creERT2 *Apc*^fl/fl^), generating *Apc*^fl/fl^ *Kras*^fl/G12D^ or *Apc*^fl/fl^ *Kras*^+/G12D^ mouse lines as simple, tractable models of oncogene-induced hyperproliferation in vivo. Homozygous deletion of *Apc* in the murine intestine results in a robust phenotype, driven by hyperproliferation and altered differentiation of the intestinal crypt epithelium^29^. Moreover, we have shown that this phenotype is exacerbated by expression of oncogenic *Kras*^10,11^. Using this system, we investigated whether loss of wild-type *Kras*, in the context of an oncogenic *Kras*^G12D^ mutation and *Apc* loss, impacted epithelial proliferation in vivo. Indeed, this was the case with significantly enhanced proliferation, as denoted by BrdU incorporation, observed in the intestinal epithelium of *Apc*^fl/fl^ *Kras*^fl/G12D^ mice when compared to *Apc*^fl/fl^ *Kras*^+/G12D^ mice (Fig. 2a, b), with the area of proliferative cells extending higher in the villus epithelium in *Apc*^fl/fl^ *Kras*^fl/G12D^ mice. This was not due to *Kras* copy number or allele changes as confirmed using droplet PCR (Supplementary Fig. 2a, b). The ectopic proliferation/dedifferentiation of cells in the villus epithelium is a key feature of *Kras*^G12D^ mutation in the context of *Apc* deficiency, and is concomitant with an acquired ability of mutant cells to form organoid cultures in vitro^11^. Consistent with the observed proliferation in the villus epithelium of *Apc*^fl/fl^ *Kras*^fl/G12D^ mice, the characteristic organoid forming capacity was enhanced when compared to *Apc*^fl/fl^ *Kras*^+/G12D^ mice (Supplementary Fig. 2c, d). These data indicate that loss of the wild-type copy of *Kras* in the context of concomitant oncogenic *Kras*^G12D^ mutation and *Apc* depletion can enhance a number of *Kras*^G12D^ associated phenotypes.

We hypothesised that loss of the wild-type *Kras* allele may alter baseline gene dosage of the oncogenic mutant allele, and as a result, lead to enhanced KRAS activation and increased signalling flux through downstream effector pathways and any associated feedback loops. This hypothesis was tested through comparison of organoid cultures derived from *Apc*^fl/fl^ *Kras*^fl/G12D^ and *Apc*^fl/fl^ *Kras*^+/G12D^ intestinal tissues. Initially, we quantified the relative proportion of GTP-bound, active RAS proteins. In these pull-down assays, *Apc*^fl/fl^ *Kras*^fl/G12D^ organoids were characterised by enhanced binding of RAS to the RAS-binding domain (RBD) of BRAF, when compared to *Apc*^fl/fl^ *Kras*^+/G12D^, suggestive of a larger pool of active RAS (Fig. 2c, d). It is well known that increased RAS activity translates into increased activation of downstream effector pathways such as the MAPK cascade^30^. To determine whether MAPK activity was increased in *Apc*^fl/fl^ *Kras*^fl/G12D^ and *Apc*^fl/fl^ *Kras*^+/G12D^ organoids, we quantified expression of known ERK-regulated transcripts, finding these to be enriched in *Apc*^fl/fl^ *Kras*^fl/G12D^ organoids (Fig. 2e). To evaluate MAPK activity in more depth, we assessed downstream effector activity in these lines through quantitative immunoblotting. We detected a clear increase in phosphorylation of ERK1/2 and MEK1/2 in *Apc*^fl/fl^ *Kras*^fl/G12D^ organoids, with phosphorylation of AKT and expression of PTEN also elevated (Fig. 2f). These data indicate that loss of the wild-type copy of *Kras* enhances the relative activity of the oncogenic mutant allele and drives downstream effector pathway signalling.

**Fig. 3:**
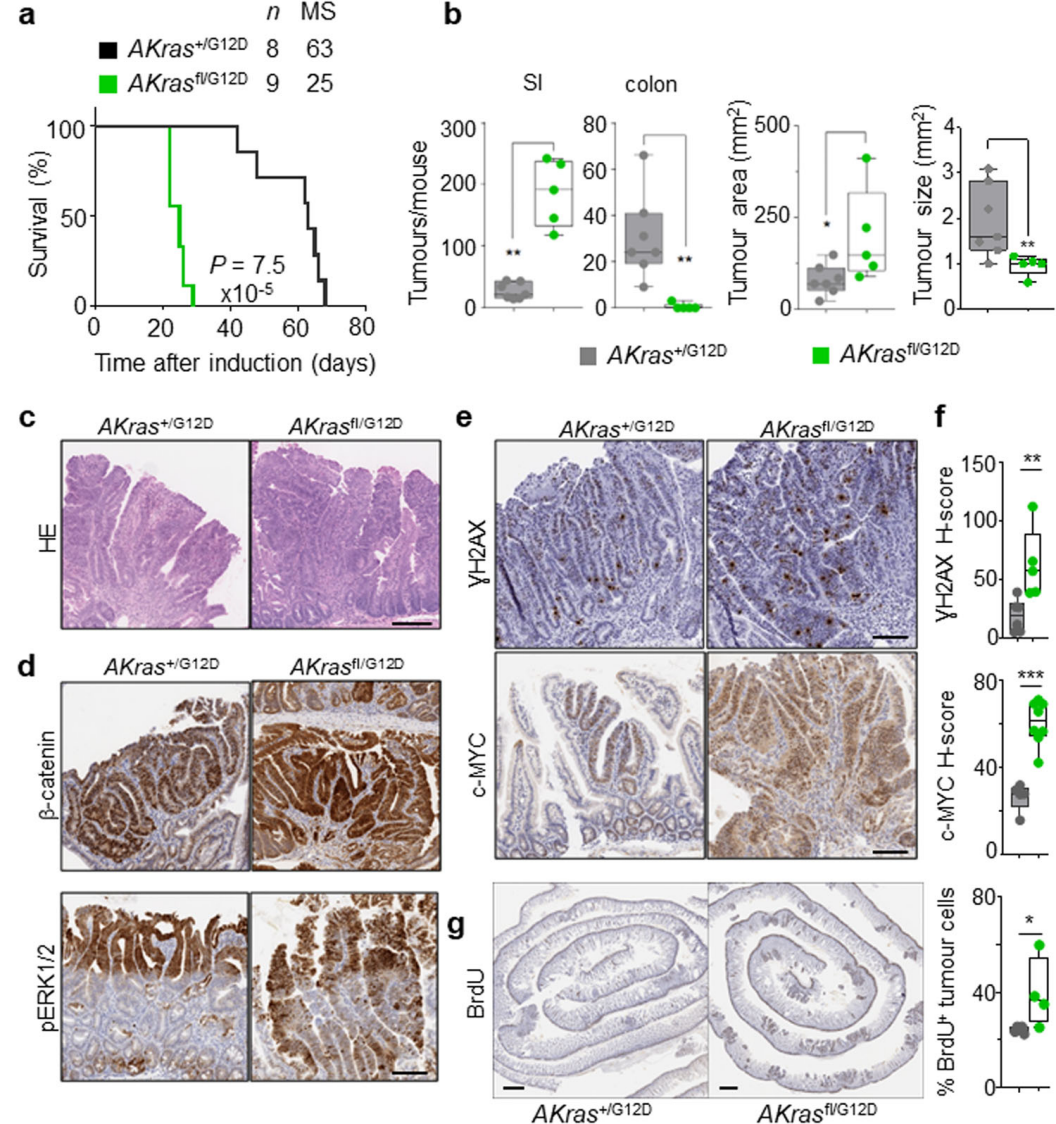
Loss of wild-type *Kras* increases mutant KRAS-driven tumourigenesis together with *Apc* loss. **a** Kaplan–Meier survival curve of villin-creERT2 *Apc*^+/fl^ *Kras*^+/G12D^ (*AKras*^+/G12D^) and villin-creERT2 *Apc*^+/fl^ *Kras*^fl/G12D^ (*AKras*^fl/G12D^) mice aged until clinical endpoint (*Apc*^+/fl^ *Kras*^+/G12D^, *n* = 8, 5M, 3F, MS, median survival = 63; *Apc*^+/fl^ *Kras*^fl/G12D^, *n* = 9, 5M, 4F, MS, median survival = 25), ****P = 7.5 × 10^-5^, log-rank (Mantel-Cox) test. **b** Left: Boxplots showing total number of tumours from *Apc*^+/fl^ *Kras*^+/G12D^ and *Apc*^+/fl^ *Kras*^fl/G12D^ mice aged until clinical endpoint in SI and Colon. Right: Boxplots showing tumour area (mm^2^) and tumour size (mm^2^) in *Apc*^+/fl^ *Kras*^+/G12D^ and *Apc*^+/fl^ *Kras*^fl/G12D^ mice aged until clinical endpoint. Boxes depict interquartile range, central line indicates median and whiskers indicate minimum/maximum values (*Apc*^+/fl^ *Kras*^+/G12D^, *n* = 7, 5M, 2F; *Apc*^+/fl^ *Kras*^fl/G12D^ *n* = 5, 3M, 2F). **P = 0.0013 (SI), **P = 0.0013 (colon), *P = 0.036 (area), **P = 0.0088 (size), one-way Mann–Whitney *U* test. **c** Representative H&E images of *AKras*^+/G12D^ (*n* = 5, 2M, 3F) and *AKras*^fl/G12D^ (*n* = 8, 5M, 3F) tumour. Dashed box highlights selected area shown in high magnification. **d** Representative images of nuclear β-catenin and pERK1/2 staining in *Apc*^+/fl^ *Kras*^+/G12D^ (*n* = 5, 2M, 3F) and *Apc*^+/fl^ *Kras*^fl/G12D^ (*n* = 8, 5M, 3F) tumours at clinical endpoint. Scale, 100 µm. **e** Representative images of γH2AX and c-MYC staining in *Apc*^+/fl^ *Kras*^+/G12D^ (*n* = 5, 2M, 3F) and *Apc*^+/fl^ *Kras*^fl/G12D^ (*n* = 8, 5M, 3F) tumours at clinical endpoint. Scale, 100 µm. **f** H-score of γH2AX (*Apc*^+/fl^ *Kras*^+/G12D^, *n* = 6, 4M, 2F; *Apc*^+/fl^ *Kras*^fl/G12D^ *n* = 5, 3M, 2F) and c-MYC (*Apc*^+/fl^ *Kras*^+/G12D^, *n* = 5, 2M, 3F; *Apc*^+/fl^ *Kras*^fl/G12D^ *n* = 8, 5M, 3F) IHC staining of (**e**). Boxes depict interquartile range, central line indicates median and whiskers indicate minimum/maximum values. **P = 0.0043, ***P = 0.0008, one-way Mann–Whitney *U* test. **g** Representative BrdU staining of *Apc*^+/fl^ *Kras*^+/G12D^ and *Apc*^+/fl^ *Kras*^fl/G12D^ small intestinal tumours at clinical endpoint. Scale, 100 µm. Right: quantification of BrdU positivity in tumour cells. Boxes depict interquartile range, central line indicates median and whiskers indicate minimum/maximum values. (*Apc*^+/fl^ *Kras*^+/G12D^, *n* = 5, 3M, 2F; *Apc*^+/fl^ *Kras*^fl/G12D^ *n* = 4, 2M, 2F). *P *=* 0.0317, one-way Mann–Whitney *U* test. Source data are provided as a Source Data file.

Given the robust impact of wild-type *Kras* deletion in the acute setting above, we next addressed the role of wild-type KRAS on oncogenic KRASG12D-driven intestinal tumorigenesis. To this end, we generated villin-creERT2 *Apc*^fl/+^ *Kras*^+/G12D^ (henceforth *AKras*^+/G12D^) and villin-creERT2 *Apc*^fl/+^ *Kras*^fl/G12D^ (henceforth *AKras*^fl/G12D^). In this setting, intestinal tumour development occurs following sporadic loss of the second copy of *Apc* in individual intestinal crypts. Targeted mutation in the intestinal epithelium was induced through intraperitoneal administration of tamoxifen, with deletion of wild-type *Kras* in the context of oncogenic *Kras*^G12D^ (*AKras*^fl/G12D^) resulting in a significant acceleration of tumorigenesis, and a consequent reduction of median survival based upon an endpoint defined by clinical signs associated with tumour burden. (Fig. 3a). The reduced time to onset of signs associated with intestinal tumorigenesis in *AKras*^fl/G12D^ mice was coincident with a striking tumour initiation phenotype, with development of numerous small lesions observed principally in the small intestine (Fig. 3b). The initiating lesions observed in *AKras*^fl/G12D^ mice were histopathologically comparable to those observed in *AKras*^+/G12D^ mice (Fig. 3c), and as expected, were positive for nuclear β-catenin (Fig. 3d). Immunohistochemical analysis demonstrated nuclear accumulation of phosphorylated ERK1/2 (Fig. 3d), increased expression of c-MYC and increased abundance of γH2AX in *AKras*^fl/G12D^ tumours when compared to *AKras*^+/G12D^ tumours, suggestive of MAPK pathway activation, increased cellular proliferation and activation of DNA damage response pathways (Fig. 3e, f). Indeed, using BrdU incorporation as a marker for cellular proliferation, we demonstrated that the tumour epithelium of *AKras*^fl/G12D^ mice is markedly more proliferative than that of *AKras*^+/G12D^ mice (Fig. 3g). Together, these results show that loss of the wild-type copy of *Kras* increases activity and signalling of the oncogenic mutant allele driving tumour initiation.

### Loss of wild-type KRAS restores sensitivity to MEK inhibition in colorectal tumours in vivo

**Fig. 4:**
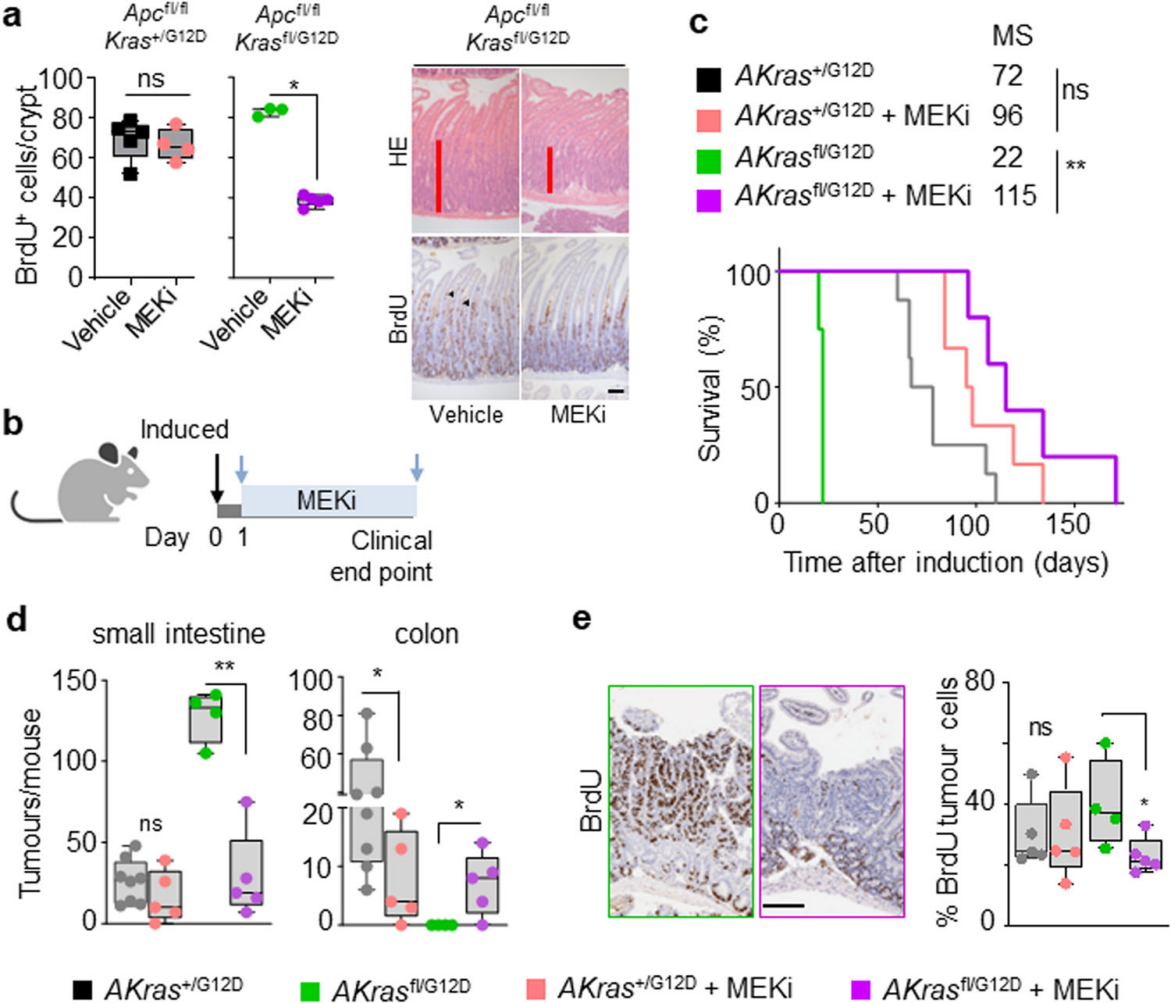
Lack of wild-type *Kras* increases sensitivity to MEK inhibition in of KRASG12D colorectal tumours in vivo. **a** Left, quantification of BrdU-positive cells per half crypt in *Apc*^fl/fl^ *Kras*^+/G12D^ and *Apc*^fl/fl^ *Kras*^fl/G12D^ mice 3 days post-induction treated with Vehicle or MEKi (AZD6244) as indicated (*Apc*^fl/fl^ *Kras*^+/G12D^ Vehicle, *n* = 5, 1M, 4F; MEKi, *n* = 4, 1M, 3F; and *Apc*^fl/fl^ *Kras*^fl/G12D^ Vehicle, *n* = 3, 1M, 2F; MEKi, *n* = 5, 3M, 2F). *P = 0.017. Right, representative H&E and BrdU images of *Apc*^fl/fl^ *Kras*^fl/G12D^ 3 days post-induction treated with Vehicle or MEKi (AZD6244) as indicated. Arrowheads show BrdU^+ve^ hyper proliferative cells. Experiments carried out on C57BL/6J background ≥ N2. **b** Schematic presenting experimental approach. *Apc*^+/fl^ *Kras*^+/G12D^ and *Apc*^+/fl^ *Kras*^fl/G12D^ mice treated with MEKi one day post-induction and treated to clinical endpoint. Created with BioRender.com. **c** Kaplan–Meier survival curve of *Apc*^+/fl^ *Kras*^+/G12D^ and *Apc*^+/fl^ *Kras*^fl/G12D^ mice treated as shown in (**b**) aged until clinical endpoint (*Apc*^+/fl^ *Kras*^+/G12D^, *n* = 8, 2M, 6F, MS, median survival = 72; *Apc*^+/fl^ *Kras*^+/G12D^ MEKi, *n* = 6, 2M, 4F, MS, median survival = 96; *Apc*^+/fl^ *Kras*^fl/G12D^, *n* = 4, 2M, 2F, MS, median survival = 22; *Apc*^+/fl^ *Kras*^fl/G12D^ MEKi, *n* = 5, 4M, 1F, MS, median survival = 115). **P = 0.0050, ns not significant, log-rank (Mantel-Cox) test. **d** Boxplots showing total number of tumours from *Apc*^+/fl^ *Kras*^+/G12D^ and *Apc*^+/fl^ *Kras*^fl/G12D^ mice untreated or treated with MEKi as indicated in (**c**) and aged until clinical endpoint in SI and Colon (*Apc*^+/fl^ *Kras*^+/G12D^, *n* = 8, 2M, 6F; *Apc*^+/fl^ *Kras*^+/G12D^ MEKi, *n* = 5, 2M, 3F; *Apc*^+/fl^ *Kras*^fl/G12D^, *n* = 4, 2M, 2F; *Apc*^+/fl^ *Kras*^fl/G12D^ MEKi, *n* = 5, 4M, 1F). **P = 0.0079 (*Apc*^+/fl^ *Kras*^fl/G12D^ SI), *P = 0.0281, *P = 0.0397 (colon). **e** Left: Representative BrdU images of *Apc*^+/fl^ *Kras*^+/G12D^ and *Apc*^+/fl^ *Kras*^fl/G12D^ tumours from mice treated with MEKi from day 1 post-induction until clinical endpoint. Right: boxplot showing percentage of BrdU-positive tumour cells in *Apc*^+/fl^ *Kras*^+/G12D^ and *Apc*^+/fl^ *Kras*^fl/G12D^ treated with MEKi (*Apc*^+/fl^ *Kras*^+/G12D^ vehicle, *n* = 5, 1M, 2F; *Apc*^+/fl^ *Kras*^+/G12D^ MEKi, *n* = 5, 2M, 3F; *Apc*^+/fl^ *Kras*^fl/G12D^ untreated, *n* = 4, 2M, 2F; *Apc*^+/fl^ *Kras*^fl/G12D^ MEKi, *n* = 5, 4M, 1F), *P = 0.0159. **a**, **d** and **e** Boxes depict interquartile range, central line indicates median and whiskers indicate minimum/maximum values, one-way Mann–Whitney *U* test. Scale, 100 μm. Source data are provided as a Source Data file.

We have demonstrated that altered allelic balance of oncogenic *Kras*^G12D^ has a substantial impact upon tumour initiation in models of intestinal disease. Given that *KRAS* mutation is clinically associated with resistance to targeted therapies, we next investigated whether therapeutic efficacy is positively or negatively influenced by allelic imbalance at the *Kras* locus. We have previously shown that the observed intestinal crypt epithelium hyperproliferation characteristic of the *Apc*^fl/fl^ *Kras*^+/G12D^ model is resistant to MEK inhibition^11,31^, and that this proliferation is enhanced in *Apc*^fl/fl^ *Kras*^fl/G12D^ mice concomitant with increased KRAS and MAPK activity (Fig. 2c, f). We reasoned that this increased MAPK activation might result in an acquired sensitivity to inhibition of MEK1/2 with a clinically relevant targeted therapeutic agent (AZD6244/selumetinib). As previously, we found that treatment of *Apc*^fl/fl^ *Kras*^+/G12D^ mice with AZD6244 had no impact upon intestinal hyperproliferation. Importantly, and in contrast, treatment with AZD6244 not only significantly decreased proliferation in the intestinal crypt epithelium of *Apc*^fl/fl^ *Kras*^fl/G12D^ mice but suppressed proliferation to a level below that of vehicle-treated *Apc*^fl/fl^ *Kras*^+/G12D^ mice (Fig. 4a). We next tested whether MEK1/2 inhibition had a similar suppressive effect upon the process of intestinal tumourigenesis. To do this, we treated *AKras*^fl/G12D^ or *AKras*^+/G12D^ mice with AZD6244 (25mgkg^-1^, BID) from 1-day post-induction of genetic recombination (Fig. 4b). We found that inhibition of MEK1/2 had a modest impact on the survival of *AKras*^+/G12D^ mice, based upon endpoint defined by onset of clinical signs associated with tumour burden, but significantly extended survival in *AKras*^fl/G12D^ mice (median survival extended from 26 days to 115 days) (Fig. 4c). This extension in survival was accompanied by a dramatic reduction in the number of small intestinal tumours (Fig. 4d) alongside increase in colonic tumour number, albeit at greatly increased time post-induction. A significant reduction in tumour cell proliferation (BrdU incorporation) was also observed in *AKras*^fl/G12D^ mice compared to *AKras*^+/G12D^ derived tumours (Fig. 4e). These data suggest that wild-type *Kras* acts to suppress the penetrance of mutant oncogenic *Kras*^G12D^, thus dampening MAPK signalling and contributing to therapeutic resistance in *Kras* mutant tumours, a key clinical problem.

### Loss of wild-type KRAS potentiates oncogenic KRASG12D driven tumorigenesis in the absence of exogenous WNT mutations in vivo

**Fig. 5:**
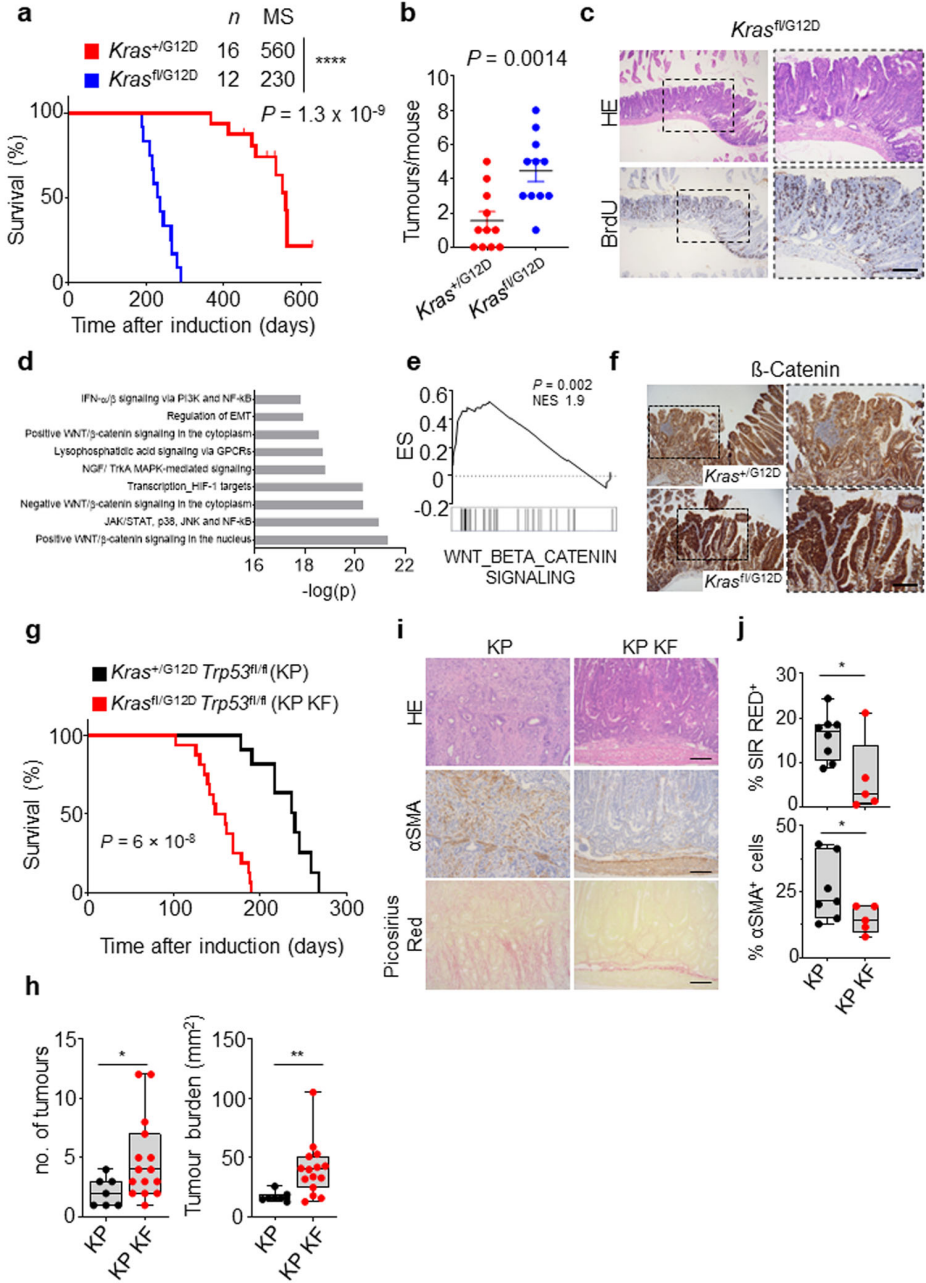
Wild-type *Kras* deletion increases tumour initiation and alters progression of KRASG12D mutant colorectal tumours following loss of p53. **a** Kaplan–Meier survival curve of villin-creERT2 *Kras*^+/G12D^ and *Kras*^fl/G12D^ mice aged until clinical endpoint (*Kras*^+/G12D^, *n* = 16, 5M, 11F; *Kras*^fl/G12D^, *n* = 12, 4M, 8F). ****P = 1.3 × 10^-9^. MS, median survival. **b** Dotplot showing total number of tumours from *Kras*^+/G12D^ and *Kras*^fl/G12D^ 2D mice. Data are mean ± s.e.m. (*Kras*^+/G12D^, *n* = 11, 3M, 8F; *Kras*^fl/G12D^, *n* = 11, 4M, 7F). **P = 0.0014. **c** Representative H&E and BrdU IHC images of *Kras*^fl/G12D^ mice. Representative of 6 biological replicates per genotype. Dashed boxes highlight selected areas shown in high magnification. Scale, 200 µm. **d** Metacore Network analysis of differentially expressed genes from *Kras*^fl/G12D^ tumours. **e** Geneset enrichment plot for Wnt beta-catenin signalling signature from the ‘Hallmark’ geneset collection of tumours derived from *Kras*^fl/G12D^ mice. *X*-axis shows normalised enrichment score (NES), and the *P* value (computed and corrected for multiple testing using the Benjamini–Hochberg procedure). **f** Representative β-catenin IHC staining in *Kras*^+/G12D^ and *Kras*^fl/G12D^ mice. Representative of 5 biological replicates per genotype. Dashed boxes highlight selected areas shown in high magnification. Scale, 200 µm. **g** Kaplan–Meier survival curve of *Kras*^+/G12D^ *Trp53*^fl/fl^ (KP) and *Kras*^fl/G12D^ *Trp53*^fl/fl^ (KP KF) mice aged until clinical endpoint (KP, *n* = 10, 7M, 3F; KP KF, *n* = 16, 6M, 10F) ****P = 6 × 10^-8^. **h** Boxplots showing total number of tumours (left) and tumour burden, mm^2^ (right) in KP and KP KF mice (KP, *n* = 7, 2M, 5F, KP KF, *n* = 15, 6M, 9F). *P = 0.02, **P = 0.0017. Please note control KP cohort used in (**g**) and (**h**) comprise different mice. **i** Representative H&E, αSMA (alpha-smooth muscle actin) and Sirius Red staining of KP and KP KF tumours. Representative of KP *n* = 8; KP KF *n* = 5. Scale, 100 µm. **j** Boxplots showing Sirius Red positivity (%) and αSMA positive cells (%) of KP and KP KF tumours (KP *n* = 7; KP KF, *n* = 5). *P = 0.0326 (Sirius Red), *P *=* 0.024 (αSMA). **h** and **j** *boxes* depict interquartile range, central line indicates median and whiskers indicate minimum/maximum values. *P* values in **a** and **g** are log-rank (Mantel-Cox) tests. **b, h** and **j** are one-way Mann–Whitney U tests. Source data are provided as a Source Data file.

Within the intestinal epithelium KRAS mutation alone is not sufficient to drive tumourigenesis. Indeed, in human, KRAS mutant clones are known to commonly arise with age, in the intestinal epithelium in morphologically normal crypts. This is recapitulated by murine models where expression of an oncogenic *Kras*^G12D^ mutant allele in isolation throughout the intestinal epithelium (villin-creERT2 *Kras*^+/G12D^) results in lowly penetrant intestinal lesion development, which translates to an overall survival of greater than 12 months. Given the impact of combined deletion of wild-type *Kras* and expression of oncogenic *Kras*^G12D^ on both intestinal homoeostasis, and tumour initiation in the context of heterozygous loss of *Apc*, we next asked whether these alterations in *Kras* allelic balance could drive tumour initiation in isolation. Here, we induced genetic recombination in villin-creERT2 *Kras*^fl/G12D^ or villin-creERT2 *Kras*^+/G12D^ mice, and assessed survival based upon sampling due to onset clinical signs associated with significant intestinal tumour burden. As expected, villin-creERT2 *Kras*^+/G12D^ alone resulted in inefficient tumour initiation, with median survival for this group at around 16 months. Strikingly, mice in the villin-creERT2 *Kras*^fl/G12D^ group developed tumours much more rapidly than the control group, exhibiting a greatly reduced median survival of around 230 days/9 months (Fig. 5a). The tumours from both *Kras*^fl/G12D^ and *Kras*^+/G12D^ mice were predominantly found in the small intestine, and were characteristically large adenomas. However, tumour number was significantly increased in the small intestine of *Kras*^fl/G12D^ mice (Fig. 5b, c). This demonstrates that even when the only exogenously introduced driver mutation is *Kras*^G12D^, loss of wild-type *Kras* significantly promotes intestinal tumourigenesis, which in turn translates into significantly shortened survival.

To better understand the mechanistic impact of deletion of wild-type *Kras* in the context of oncogenic *Kras*^G12D^, we transcriptionally profiled tumours arising in villin-creERT2 *Kras*^fl/G12D^ mice and compared to adjacent non-transformed tissue. Geneset enrichment analysis (GSEA), identified key oncogenic programmes associated with KRAS signalling, MEK and AKT were enriched in villin-creERT2 *Kras*^fl/G12D^ tumours (Supplementary Fig. 3a). Importantly, GSEA and Metacore analysis of differentially expressed genes showed an overrepresentation of WNT and β-catenin signalling pathways (Fig. 5d, e). Given that aberrant activation of the Wnt/β-catenin pathway, and its downstream transcriptional networks, is a common initiating event in CRC, and results from stabilisation and nuclear accumulation of the transcriptional co-activator β-catenin, we interrogated tumours arising in villin-creERT2 *Kras*^fl/G12D^ and villin-creERT2 *Kras*^+/G12D^ mice for nuclear accumulation of β-catenin. Consistent with the transcriptional enrichment of Wnt signalling pathways (Fig. 5e), tumours arising in *Kras*^fl/G12D^ intestines showed positivity for nuclear β-catenin while *Kras*^+/G12D^ intestines more predominantly exhibited membranous β-catenin (Fig. 5f).

### Loss of wild-type KRAS alters tumour progression and metastasis of aggressive mutant KRAS-driven colorectal tumours

Thus far, we have only investigated the role of wild-type KRAS in mouse models of adenoma. Recent reports have shown that changes in *KRAS* gene dosage alter the clonal evolution of tumours and change the metastatic incidence in KRAS mutant cancers^23^. Given the dramatic role we have observed in tumour initiation, we next wished to see how important wild-type KRAS would be in tumour progression. We have previously demonstrated that deletion or mutation of the tumour suppressor gene *Trp53* alongside activating mutation of *Kras* can give rise to aggressive, late-stage adenocarcinoma development in the intestine^32^. Indeed, the human paralogue, TP53 is inactivated in more than half of human colorectal cancers (TCGA). These models therefore represent an ideal system for interrogating the impact of wild-type *Kras* deletion in a more complex, aggressive, clinically relevant oncogenic KRAS^G12D^ driven disease.

To achieve this, we interbred the villin-creERT2 *Kras*^fl/G12D^ mouse line with a *Trp53* conditional knockout allele to generate villin-creERT2 *Kras*^fl/G12D^ *Trp53*^fl/fl^ (henceforth referred to as KP KF) and villin-creERT2 *Kras*^+/G12D^ *Trp53*^fl/fl^ (henceforth referred to as KP) mice. While KP mice developed on average one intestinal tumour and exhibited a median overall survival of 240 days, with very lowly penetrant metastasis (Fig. 5g, h), KP KF mice developed on average four intestinal tumours and exhibited a median overall survival of 150 days (Fig. 5g, h). Histological analysis of tumours arising in KP KF mice suggested a morphology akin to human tubulovillous adenoma, in contrast to the tumours which arose in control KP mice, which exhibit a serrated morphology. Moreover, evidence of local invasion or poor differentiation, features typically associated with advanced disease, were apparent in KP tumours but absent from KP KF tumours (Fig. 5i). In addition, there were clear differences in the stromal microenvironment of the KP KF tumours, such as a lack of infiltrating alpha-smooth muscle actin (αSMA)-positive stromal cells and low levels of stromal collagen deposition (as indicated by picrosirius red), again, contrasted by KP tumours (Fig. 5i, j). These data suggest that while loss of the wild-type copy of *Kras* in an aggressive *Kras* mutant-driven model of intestinal cancer can drive accelerated tumour initiation, it does not endow tumours with increased invasion or aggression, indeed these features appear suppressed.

**Fig. 6:**
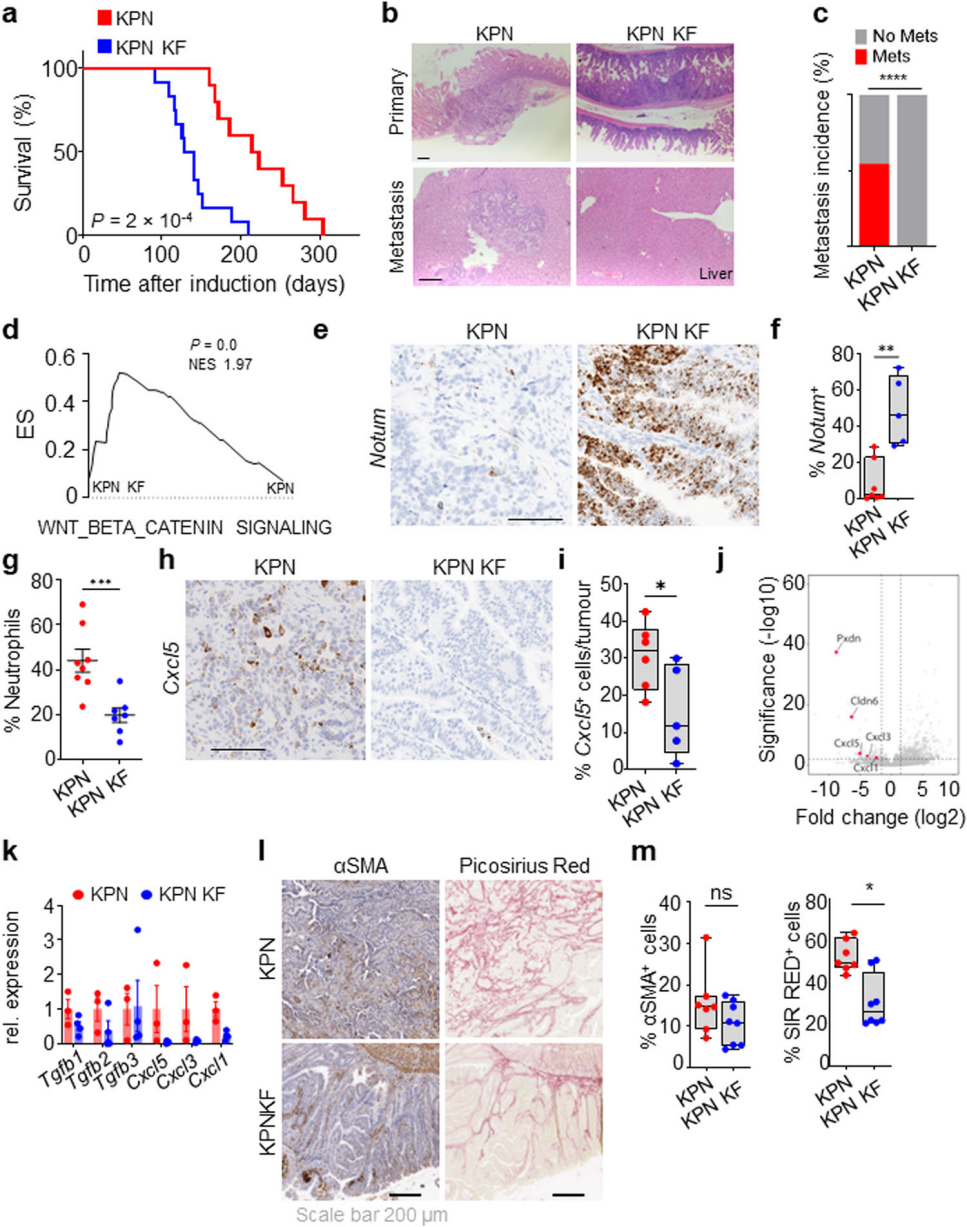
Wild-type *Kras* deletion alters progression and metastasis of KPN tumours. **a** Kaplan–Meier survival curve of *Kras*^+/G12D^ *Trp53*^fl/fl^ *Rosa26*^N1icd/+^ (KPN) and *Kras*^fl/G12D^ *Trp53*^fl/fl^ *Rosa26*^N1icd/+^ (KPN KF) mice aged until clinical endpoint (KPN, *n* = 10, 5M, 5F, KPN KF, *n* = 12, 6M, 6F) ***P = 2 × 10^-4,^ log-rank (Mantel-Cox) test. **b** Representative H&E images of primary tumour and metastasis (liver) of mice from (**a**). Scale, 200 μm. **c** Incidence of metastasis (%) in KPN and KPN KF mice aged until clinical endpoint (KPN, *n* = 11, 6M, 5F; KPN KF, *n* = 11, 5M, 6F) ****P = 1 × 10^-15,^ two-tailed chi-square test. **d** GSEA of hallmark WNT-β-Catenin signalling in KPN KF tumours compared to KPN. *X*-axis shows normalised enrichment score (NES), and the *P* value (computed and corrected for multiple testing using the Benjamini–Hochberg procedure). **e** Representative *Notum* in situ hybridisation (ISH) in KPN and KPN KF tumours. Scale, 100 μm. **f** Boxplots showing *Notum* (ISH) positive cells per tumour (%) of KPN and KPN KF tumours (KPN, *n* = 7, 2M, 5F; KPN KF, *n* = 5, 2M, 3F). **P = 0.0013. **g** Dotplot showing neutrophils percentage in systemic blood of KPN and KPN KF mice aged to clinical endpoint (KPN, *n* = 8, 6M, 2F; KPN KF, *n* = 7, 5M, 2F). ***P = 0.0006. **h** Representative *Cxcl5* ISH in KPN and KPN KF tumours. Representative of five mice per genotype. Scale, 100 μm. **i** Boxplots showing *Cxcl5* positive cells per tumour (%) of KPN and KPN KF tumours (KPN, *n* = 6, 2M, 4F; KPN KF, *n* = 5, 2M, 3F). *P = 0.0411. **j** Volcano plot of differentially expressed genes in organoids derived from KPN KF tumours. **k** Relative expression of *Tgfβ* ligands and chemokines from organoids derived from KPN and KPN KF tumours. Data are mean ± s.e.m. KPN, *n* = 3, 3M; KPN KF, *n* = 4, 1M, 3F. **l** Representative αSMA (alpha-smooth muscle actin) and Sirius Red staining of KPN and KPN KF tumours. Scale, 200 µm. **m** Boxplots showing Sirius Red positivity (%) and αSMA positive cells (%) of ageing or vehicle-treated KPN and KPN KF tumours (KPN *n* = 7, 2M, 5F; KPN KF *n* = 8, 4M, 4F). *P *=* 0.0103. **f**, **i** and **m** boxes depict interquartile range, central line indicates median and whiskers indicate minimum/maximum values. **f**, **g**, **i** and **m** are one-way Mann–Whitney *U* tests. Source data are provided as a Source Data file.

Considering this, we decided to further investigate the role of wild-type *Kras* in aggressive, late-stage KRAS-mutant CRC using our recently described metastatic KPN model (villin-creERT2 *Kras*^+/G12D^ *Trp53*^fl/fl^ *Rosa26*^N1icd/+^). These KPN mice develop intestinal adenocarcinoma and exhibit highly penetrant metastasis, predominantly to the liver^32^. We interbred KPN mice with KP KF mice to generate (villin-creERT2 *Kras*^fl/G12D^ *Trp53*^fl/fl^ *Rosa26*^N1icd/+^; henceforth referred to as KPN KF). Strikingly, tumorigenesis was significantly accelerated in KPN KF mice with deletion of wild-type *Kras*, reflected by a significant reduction in overall survival based upon endpoint defined by clinical signs associated with tumour burden (Fig. 6a). Using droplet PCR we confirmed this acceleration in tumorigenesis of KPN KF mice was not due to mutant *Kras* induced changes in *Kras* copy number (Supplementary Fig. 4a, b). Comparative histological analysis of tumours from KPN and KPN KF mice showed that tumours arising from KPN KF showed lack of local invasion with remarkable lack of metastasis incidence (Fig. 6b, c). To gain a better mechanistic insight into the impact of *Kras* deletion in this setting, we first performed transcriptional analysis of tumours derived from KPN KF and KPN mice. Interestingly, KPN KF tumours showed a significant enrichment of Wnt signalling pathway (Fig. 6d) and expression of the surrogate Wnt marker *Notum*, compared to KPN tumours (Fig. 6e, f). It is notable that relatively low levels of Wnt activation is a key feature of the highly metastatic KPN tumours, while comparable high-Wnt APC-deficient, APN tumours are non-metastatic^32^. Consistent with the high-Wnt activation signature in KPN KF tumours, comparative analysis of several published Wnt transcriptional signatures in tumours from APN, KPN and KPN KF mice clearly showed higher Wnt activation in KPN KF tumours (Supplementary Fig. 4c). Collectively, these data show that *Kras* mutant tumours lacking wild-type *Kras* in a KPN setting activate more robust levels of Wnt signalling during tumorigenesis. This said, it is noteworthy that primary tumours arising in the intestine of KPN KF mice are adenomatous, while those arising in KPN mice are of a more aggressive adenocarcinoma/carcinomatous morphology. While the underlying genetics will undoubtedly play a key role in defining the molecular makeup of the disease, the reduced histopathological grade of tumours found in KPN KF may directly influence immune and inflammatory infiltrate.

Nonetheless, we have previously shown that a critical feature of metastasis in the Notch1-driven KPN tumours is TGFβ pathway-mediated neutrophil infiltration^32^. However, we detected a lack of systemic circulating neutrophil accumulation in the blood of KPN KF mice (Fig. 6g). Given that chemokines such as *Cxcl5* are implicated in neutrophil attraction, we assessed expression of *Cxcl5* and found significantly reduced expression in the epithelium of KPN KF tumours (Fig. 6h, i). To determine how loss of wild-type *Kras* alters the TGFβ pathway and metastasis, we examined the transcriptome of tumour-derived KPN and KPN KF organoids. Interestingly, we found that NOTCH1-mediated gene expression of *Tgfb2* and chemokines, such as *Cxcl1, Cxcl3* and *Cxcl5*, was downregulated in KPN KF organoids (Fig. 6j, k). Moreover, GSEA analysis showed lack of TGFβ signalling in KPN KF organoids (Supplementary Fig. 4d). Furthermore, along with the low expression of neutrophil markers (Fig. 6h, i), *Tgfb2* expression was lowered in KPN KF tumours (Supplementary Fig. 4e). In addition, we observed clear differences in the stromal microenvironment of the KPN KF tumours, with significantly low stromal collagen deposition (picrosirius red), again, contrasted by collagen high KPN tumours (Fig. 6l, m).

**Fig. 7:**
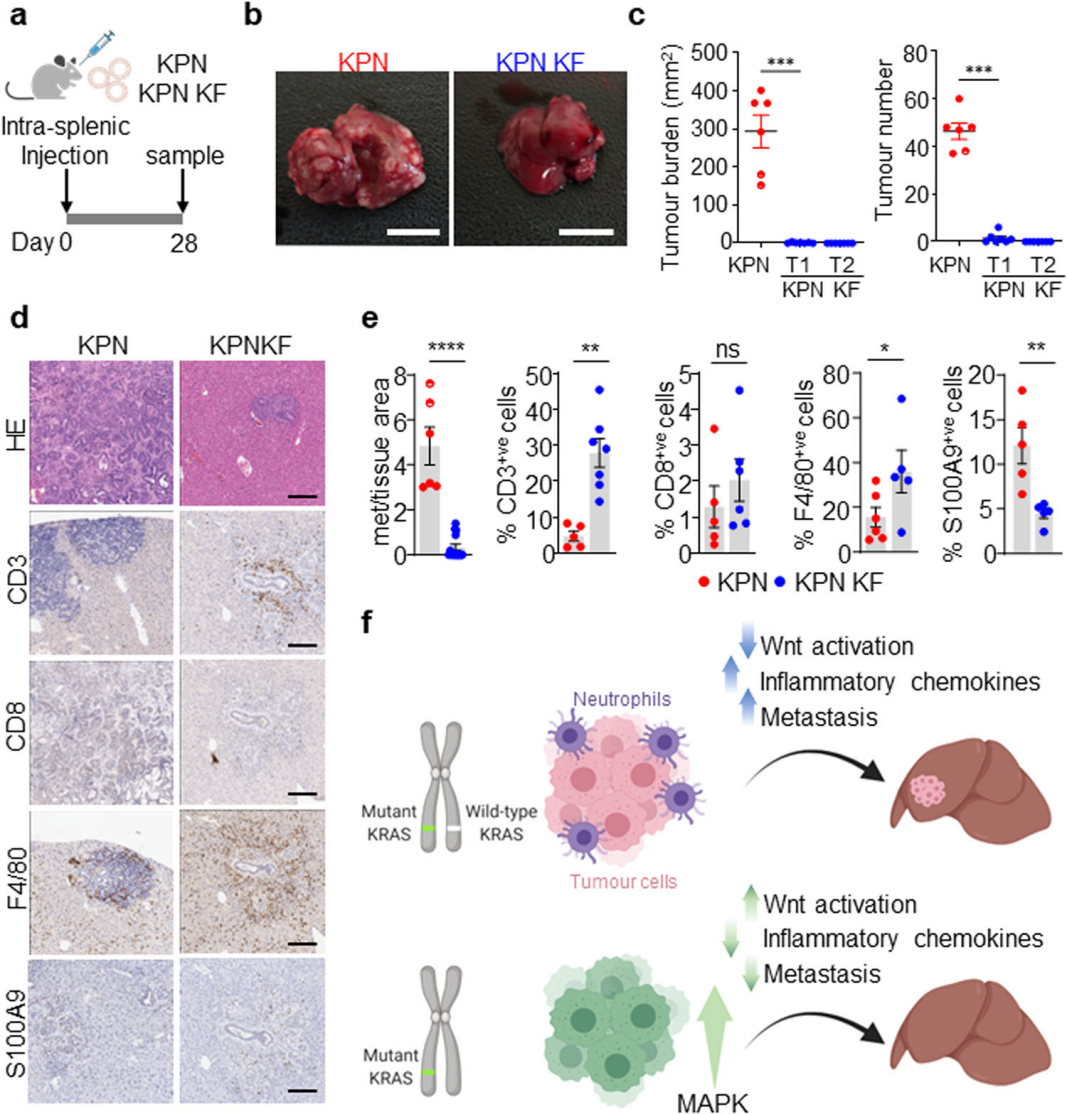
Wild-type *Kras* deficient KPN cells show reduced metastatic capacity and increased immune infiltration. **a** Schematic showing the intrasplenic transplantation of KPN and KPN KF organoids. **b** Images of liver tumour burden from KPN and KPN KF organoid transplant mice. Representative of six mice per organoid transplant. Scale, 1 cm. **c** Quantifications of liver tumour burden and tumour number from KPN and two KPN KF (T1 and T2) organoid transplants. (KPN *n* = 6, 6M, KPN KF T1 *n* = 7, 7F, KPN KF T2 *n* = 7, 7F). Data are mean ± s.e.m. Semi-circles include lung metastasis burden. ***P = 0.0006, one-way Mann–Whitney *U* test. Created with BioRender.com. **d** Representative HE, CD3, CD8, F4/80 and S100A9 IHC in KPN and KPN KF transplant mice. Representative of six mice per organoid line. Scale, 200 μm. **e** Quantifications from **d** KPN KF T1 and T2 with tumour burden represented together as KPN KF. Data are mean ± s.e.m. Representative of KPN *n* = 6, 6M, KPN KF *n* = 14, 14F, ****P = 2.5 × 10^-5^ (Met area); KPN *n* = 5, 5M, KPN KF *n* = 7, 7F, **P = 0.0013 (CD3); KPN *n* = 5, 5M, KPN KF *n* = 6, 6F, ns, *P* = 0.2143 (CD8); KPN *n* = 6, 6M, KPN KF *n* = 5, 5F, *P = 0.02 (F4/80); KPN *n* = 5, 5M, KPN KF *n* = 5, 5F, **P = 0.0079 (S100A9), one-way Mann–Whitney *U* test. **f** Schematic model showing the role of wild-type KRAS in KRAS mutant CRC. Loss of wild-type *Kras* in KPN KF tumours promotes tumour initiation with WNT activation, an enhanced immune infiltrate and blunted metastasis. Created with BioRender.com. Source data are provided as a Source Data file.

Given that we have previously shown that KPN tumour cells generate an immunosuppressive, pro-metastatic environment, we assessed the ability of KPN KF tumour cells to produce such an immunosuppressive environment to generate distant metastasis. To address this experimentally, we performed orthotopic intrasplenic transplantation of KPN and KPN KF tumour-derived organoids in syngeneic recipient mice (Fig. 7a). All mice with KPN organoid transplantation showed metastasis to either liver or lung (Fig. 7b). However, the metastasis burden was significantly blunted in KPN KF organoid transplanted mice (Fig. 7b, c). Remarkably, we saw a significant increase in immune cell infiltration in KPN KF transplants compared to the KPN control tumours (Fig. 7d, e). The observed impact upon immune and inflammatory infiltrate was independent of the size of metastatic deposits found in the liver – the decreased neutrophil and increased lymphocyte association with metastatic deposits in KPN KF transplants versus KPN transplants was maintained when analysis was restricted to tumours of comparable size (Supplementary Fig. 5a, b). Moreover, the apparent impact upon immune cell infiltration was also confirmed in the KPN KF GEMM tumours (Supplementary Fig. 4g). Together, this indicates that loss of wild-type *Kras* with epithelial NOTCH1 activation in KPN tumours activates Wnt, restricts the TGFβ mediated neutrophil recruitment required to generate an immunosuppressive, pro-metastatic niche and blunts the metastases of tumours lacking wild-type *Kras* (Fig. 7f).

## Discussion

Activating mutations in proto-oncogenes like *KRAS* are the initial events in the development of cancer followed by sequential progression involving additional genetic hits in tumour suppressor genes. Here we provide evidence that loss of wild-type *Kras* facilitates and accelerates tumour initiation, but drastically alters tumour evolution in CRC. We demonstrate that loss of wild-type *Kras* increases the dependence of these tumours on the MAPK pathway thereby making them susceptible for targeting through MEK1/2 inhibition. In addition, we find that loss of tumour suppressors like p53 together with loss of wild-type *Kras* leads to faster tumour initiation but altered progression in *Kras* mutant tumours.

The role of wild-type RAS isoforms is contradictory in tumours. While the biochemical properties and transforming potential of RAS oncoproteins suggest a dominant mechanism for these mutant proteins during tumorigenesis, several studies have argued for and against the antagonising properties of wild-type RAS in the presence of oncogenic variants^18,33,34^. There is also evidence that this is context dependent based on the RAS isoform and the tissue type under investigation^35^. Early reports suggested that presence of wild-type *Hras* or *Nras* can suppress tumourigenic phenotypes of mutant RAS encoding genes^36^. While loss of wild-type *Kras* was shown to promote activation of all RAS isoforms in a leukaemia model^37^, loss of wild-type *Nras* did not alter the tumorigenic behaviour of mutant NRAS-G12D in a hematopoietic cancer model^38^. Our genetic and functional analyses using *Kras* mutant mouse models of colorectal cancer suggest that wild-type *Kras* plays a significant role in the cancer cell fitness, evolution and therapeutic susceptibility of *Kras* mutant cells in vivo.

Recent reports have highlighted that not all *KRAS* mutations are equal, with each *KRAS* mutation having unique biochemical, signalling and functional properties in cells^5–7^. Our data suggest that these well-characterised isoform-specific differences may be further compounded by allelic changes such as loss of the wild-type allele. Recent work from Ambrogio et al. suggested that dimerization of oncogenic KRAS is essential to sustain oncogenic function and the growth inhibitory effect of wild-type KRAS is linked to its dimerization with mutant KRAS^19^. We show that wild-type *Kras* suppresses phenotypes associated with mutant *Kras* in colorectal models in vivo. While KRAS-driven CRC and PDAC are resistant to MEKi, the *KRAS* allelic configuration has been shown to modulate MEKi sensitivity in AML and CRC cell lines^1^. Our data show that loss of wild-type *Kras* causes cancer cells to amplify oncogenic signalling to optimise growth, leading them to be more dependent or addicted to certain activated pathways that become therapeutic susceptibilities. The enhanced sensitivity of *Kras*^G12D/fl^ tumours to MEKi supports this idea. Similarly, this would also suggest that in addition to screening of patients for *KRAS* mutation status, stratification of patients with *KRAS* allelic status that corresponds to selective pressure for outgrowth of tumours with increased MAPK signalling are more likely to render this subset of patients more susceptible to effector pathway inhibitors.

The idea that mutant *KRAS* gene dosage or allelic configuration can alter tumour progression has been explored recently. It is notable that as such changes are reported to be common and occur in high frequencies in *KRAS* mutant cancers, our data argue that loss of wild-type *Kras* significantly increases initiation of *Kras* mutant intestinal tumours^23,26^. In contrast to *Kras*^+/G12D^ tumours, we find that *Kras*^fl/G12D^ tumours exhibit a reduction in serration, alongside reduced invasion, differentiation, and metastasis – all features previously associated with high levels of Wnt signalling in CRC. We have previously identified TGFB2 and other chemokine secretion from the epithelium, driven by Notch activation as a key driver of metastasis in mouse models of CRC^32^. Considering the co-ordinated control of, and commonly reciprocal relationship between Wnt and Notch signalling in CRC^39^, it is intriguing that the suppression of metastasis observed in complex *Kras*^fl/G12D^ tumours occurs hand-in-hand with transcriptional enrichment of canonical Wnt-associated gene programmes. This is also consistent with reports showing that Wnt-high human CRC with elevated expression of Wnt target genes have better prognosis than Wnt-low tumours^40,41^. In the model systems reported here, Wnt-dependent transcriptional programmes were enriched in *Kras*^fl/G12D^ tumours when compared to *Kras*^+/G12D^ intestinal tumours, indicative of further crosstalk between the *Kras* and Wnt signalling pathways. When viewed in the round, these observations suggest complex interplay between the WNT, NOTCH and KRAS signalling axes in determining disease trajectory in colorectal cancer, and that allelic imbalances at the *KRAS* locus might skew this relationship, ultimately impacting prognosis and therapeutic sensitivity.

## Methods

### Mouse models and experiments

All experiments were performed according to UK Home Office regulations (Project licences 70/8646, PP3908577), under the oversight of the Animal Welfare and Ethical Review Board (AWERB) of the University of Glasgow. Mice were genotyped by Transnetyx (Cordoba, TN, USA). For all experiments, male and female mice of >20 g and between 6 and 15 weeks of age were used, unless otherwise specified. For intestinal studies, the transgenes and alleles used were as follows – villin-creERT2^42^, *Apc*^fl^ ^43^, *Kras*^LSL-G12D^ ^44^, *Braf* ^V600E(tm1Mmcm)^^45^, *Braf*^V600E(tm1Cpri)^^46^, *Trp53*^fl^ ^47^ and *Rosa26*^N1icd^ ^48^. Recombination by Villin-creERT2 was induced with one intraperitoneal (i.p.) injection of 80 mg/kg tamoxifen on day 0. Analysis of villin-creERT2-induced *Apc*^fl/fl^ *Kras*^+/G12D^ and *Apc*^fl/fl^ *Kras*^fl/G12D^ mice was at day 3 after induction. For tumourigenesis studies, villin-creERT2 *Apc*^+/fl^ (with *Kras*^+/G12D^ or *Kras*^fl/G12D^), *Kras*^+/G12D^, *Kras*^fl/G12D^, KP, KP KF, KPN and KPN KF mice were sampled when exhibiting clinical signs of ill-health indicative of substantial intestinal tumour burden (typically anaemia, hunching and/or weight loss), with overall survival recorded as the time from induction to sampling. Mice and organoids comprising the were of a C57BL/6J background, backcrossed ≥ 6 generations, unless otherwise stated. The MEK inhibitor (AZD6244 or selumetinib) was administered in a concentration of 25 mg/kg twice daily via oral gavage in a vehicle of 0.5% HPMC and 0.1% Tween-80.

Intrasplenic transplantation injections were performed as previously described^32^ using a cell suspension of primary tumour organoids derived from C57BL/6 KPN or KPN KF mice. Organoid donor and recipient mice were strain matched. Metastatic tumour burden and tumour number were scored macroscopically upon dissection, with tumour burden recorded as cumulative area of metastatic deposit observed across all liver lobules from each individual mouse. For analysis of composition of size-matched metastatic deposits, the mean area of metastatic deposit from the KPN KF cohort was calculated microscopically from a single H&E liver section per mouse. This value ± 1 × standard deviation (σ) was used as a range of tumour area (21188.95–154971.4 µm^2^), which could in turn be used to select size-matched metastatic deposits from the KPN cohort for comparative analysis.

The smallest sample size was chosen that could give a significant difference, in accordance with the 3Rs. Given the robust phenotypes of the villin-creERT2 *Apc*^fl/fl^ model, and prediction that KRAS was essential, the minimum size sample size assuming no overlap in control vs. experimental is three animals.

### Generation of Kras and Braf conditional alleles

To generate conditional alleles of *Kras* and *Braf*, embryonic stem cell lines (mESCs) were imported from EUMMCR^49^.

Briefly, for *Kras* allele, the imported mESC was HEPD0073_1_E09 (JM8.F6 ES cells). This cell line carries *Kras*^tm1a(EUCOMM)Hmgu^, a knockout of the first allele of the *Kras* gene (Ensembl ID: ENSMUSG00000030265 in Genome Assembly GRCm39). This allele places loxP sites on either side of exon 3 (ENSMUSE00000545805) of the mouse *Kras* gene transcript (Ensembl Transcript ID: Kras-201; ENSMUST00000032399.12). The presence of the isolated 3′ loxP site was confirmed by PCR using 5′-GTT ACA GCA GTT ACA TGA CTT GTC C-3′ and 5′-TTA AGG TCA TTG GAG TAA CAC CAT C-3′ (212bp). Following confirmation of correctly targeted clone for the *Kras* allele, mouse lines were derived by injection of targeted mESCs cells into Balb/c blastocysts according to standard protocols^50^. Chimeras were identified by black coat colour.

For *Braf*, the mESC imported was EPD0608_5_D04 (JM8A3.N1 ES cells). This cell line carries *Braf*^tm1a(EUCOMM)Wtsi^, a knockout of the first allele of the *Braf* gene (Ensembl ID: ENSMUSG00000002413 in Genome Assembly GRCm39). The allele places loxP sites on either side of exon 5 (ENSMUSE00000618030) of *Braf* gene transcript (Ensembl Transcript ID: *Braf*-201; ENSMUST00000002487.15). The presence of the isolated 3′ loxP site was confirmed by PCR using 5′- GTT CGA GAC AGT CTA AAG AAA GCA C-3′ and 5′- GGC ATT TGT CAT AGG AAT AAA CAA C-3′ (513bp). Following confirmation of correctly targeted clone for the *Braf* allele, mouse lines were derived by injection of targeted mESCs cells into C57BL/6J blastocysts according to standard protocols^50^. Chimeras were identified by agouti coat colour.

After breeding of the Kras and Braf chimeras, germline offspring were identified by coat colour and the presence of the modified allele was confirmed with the allele-specific 3′ loxP primers described above. To generate conditional alleles (tm1c) from the original knockout first allele (tm1a), both strains were subsequently crossed with a mouse strain expressing FLPe (Tg(ACTFLPe)9205Dym) to delete the selectable markers by recombination at the FRT sites^49^. Correct deletion of the selectable marker was confirmed by PCR across the remaining FRT site, using the oligos for Kras: 5′- ATT CAC AGT GTT GTG TGA CCA TTA G-3’ and 5′- AGT GAG ACC TTG TCT TAA TGC ACT C-3′ (432bp); and for Braf 5′- ACA CTC AGC TTT TTA GTG GGT ACT G-3′ and 5′- TTC CAC CAC TGA AAA TAC TTA AAG G-3′ (486bp).

Following successful validation of the mouse strains carrying the *Kras* and *Braf* conditional alleles, genotyping was subsequently carried out by a commercial genotyping service provider (Transnetyx).

### Immunohistochemistry (IHC) and RNAscope

Haematoxylin-and-eosin (H&E) staining was performed using standard protocols. Immunohistochemistry - IHC for BrdU (1:200, BD Biosciences #347580), β-catenin (1:50, BD Biosciences #610154), γ-H2AX (1:50, Cell Signalling Technologies #9718), phospho-ERK1/2 (1:400, Cell Signalling Technologies #9101), c-MYC (1:200 Abcam #ab32072), Lysozyme (1:300, Dako/Agilent #A099), αSMA (1:25000, Sigma-Aldrich #A2547), CD3 (1:100, Abcam #ab16669), CD4 (1:500, eBisoscience #14-9766-82), CD8 (1:500, eBisoscience #14-0808-82), F4/80 (1:200, Abcam #ab6640) and S100A9 (1:1500, CST #73425) was performed on formalin-fixed intestinal sections using standard protocols. Secondary antibodies used were EnVision anti-rabbit (undiluted, DAKO #K4003) and anti-mouse (undiluted, DAKO #K4001). All antibodies were commercially sourced, and validated by the manufacturer. In situ hybridisation (ISH) (RNAscope) was performed according to the manufacturer’s protocol (Advanced Cell Diagnostics RNAscope 2.0 High Definition–Brown) for *Notum, Cxcl5* and *Tgfb2*. Sections were counterstained with hematoxylin and coverslipped using DPX mountant (CellPath, UK). Picrosirius Red staining technique was used to stain collagen within tissue sections^32^.

### Assaying proliferation in vivo

Proliferation levels were assessed by measuring BrdU incorporation. Mice were injected with 250 µl of BrdU (Amersham Biosciences) 2 h before being sacrificed. IHC staining for BrdU was then performed using an anti-BrdU antibody. For each analysis, 25 half-crypts were scored from at least three mice of each genotype.

### Cell lines and crypt culture

Intestinal organoids were generated in-house from genetically engineered mouse models described above, with genotyping information provided by Transnetyx (Cordoba, TN, USA). Briefly, small intestinal and colon lines were generated from mice 3 days post tamoxifen induction. Small intestine and colon were scraped with a glass cover slip and washed repeatedly with cold PBS. Following 30 min EDTA incubation at 4 °C, crypts were isolated from tissue through mechanical pipetting, strained and cell pellets subsequently cultured in Matrigel and Advanced Dulbecco’s Modified Eagle Medium/Ham’s F-12 (Advanced DMEM/F-12, Gibco #12634010) supplemented with 2 mM L-glutamine (Gibco #A2916801), HEPES (Gibco #15630080) and 1% Penicillin-Streptomycin (Gibco #15070063). For tumour culture, a central piece of intestinal tumour was removed on dissection, washed in cold PBS and fragmented. Fragments were incubated in DNase and trypsin for 30 min and thoroughly dissociated. Advanced DMEM/F-12 media was added and the suspension pelleted. Following resuspension and straining cell pellets were cultured in Matrigel as above.

### ddPCR

Genomic DNA was extracted from snap-frozen cell pellets or mouse tissues using the QIAGEN DNeasy Blood and Tissue Kit. Reactions were performed with ddPCRSupermix and primers and probes (Bio-Rad) listed below. ddPCR was carried out according to Bio-Rad’s protocol. Droplets were generated on Bio-Rad’s QX200 with droplet generation oil, subjected to amplification (95 °C 10 min, 94 °C 30 s, 59 °C 1 min, repeated 40×, 98 °C 10 minutes, 8 °C hold), and read on Bio-Rad’s QX200 Droplet Reader running QuantaSoft software. Primer sequences are as follows: KRAS_G12D Forward: CTGCTGAAAATGA-CTGAGTA, Reverse: ATTAGCTGTATCGTCAAGG, Probe:TGGAGCTGATGGCGT with FAM fluorophore; KRAS_wt Forward: CTGCTGAAAATGA-CTGAGTA, Reverse: ATTA GCTGTATCGTCAAGG, Probe Sequence: TGGAGCTGGT-GGCG with HEX fluorophore.

### RAS-activation assay

Organoids were plated two days prior to the assay in Matrigel. For starvation, organoids were washed with PBS and fed ADF without EGF was for 30 min. After starvation organoids were quickly washed with ice-cold PBS, collected and disrupted prior to cell lysis. The following steps were performed using the Cytoskeleton RAS-activation assay biochem kit (#BK008) according to the manufacturer’s instructions.

### RNA extraction, qRT-PCR and RNA-sequencing

RNA was extracted from whole tissue, organoids or tumour tissues using QIAGEN RNAeasy kit (Cat no. 74104) according to the manufacturer’s instructions. 1 µg of total RNA was reverse transcribed to cDNA using a High-Capacity cDNA Reverse Transcription Kit (Thermo Scientific, Cat 4368814). qPCR was performed in duplicates for each biological replicate in a 20 µl reaction, with 10 µl of 2X DyNAmoHSmaster mix (Thermo Scientific, Cat no. F410L), 2 µl cDNA and 0.5mM of each of the primers. The reaction mixture without a template was run in duplicate as a control. *Gapdh* was used to normalise for differences in RNA input. For RNA sequencing, RNA integrity was analysed using NanoChip (Agilent RNA 6000 Nano kit, 5067-1511). A total of 2 µg RNA was purified by poly(A) selection. The libraries were run on the Illumina NextSeq 500 using the High Output (75 cycles) kit (2 × 36 cycles, paired-end reads, single index). Analysis of RNA-seq data was carried out as previously described^51^. Geneset enrichment analysis was performed using GSEA version 4.1.0 software (Broad Institute).

### SDS-PAGE and western blotting

Organoid pellets were washed in ice-cold PBS and lysed in RIPA buffer. Protein concentration was determined using BCA protein assay (Thermo scientific # 23225). 20ug protein were separated on a 4–12% gradient pre-cast gel (Novex #NP0322PK2) in MOPS running buffer. Samples were transferred onto PVDF membrane (Millipore, #IPFL00010). Primary antibodies were diluted as indicated in 5% BSA/TBS-T (Sigma #A9647) and incubated overnight at 4 °C (KRASG12D 1:1000, CST #14429; ß-actin 1:2000, SIGMA #A2228, ERK1/2 1:1000, CST #4695; pERK1/2 1:1000, CST #9101, MEK1/2 1:1000, CST ##8727 pMEK1/2 1:1000, CST #2338; AKT 1:1000, CST #9272; pAKT (Ser473) 1:2000, CST #4060, PTEN 1:1000, CST #9559). Secondary antibody (goat α-rabbit HRP, Dako #P0448, goat α-mouse HRP Dako #P0447) was diluted 1:2000 in 5% BSA/TBS-T and incubated for 1h at RT. All antibodies were commercially sourced, and validated by the manufacturer.

### Statistics

Statistical analyses were performed using GraphPad Prism (v8) software (La Jolla, CA, USA). Statistical comparisons for survival data were performed using Mantel-Cox (Log-Rank) test. Mann–Whitney-*U* tests were performed using GraphPad Prism. In each figure legend, the data and errors are shown, and the relevant statistical test is specified.

### Reporting summary

Further information on research design is available in the Nature Portfolio Reporting Summary linked to this article.

### Supplementary information

Supplementary Information

Reporting Summary

### Source data

Source Data

## Data Availability

RNA sequencing datasets are archived and publicly available at GEO (https://www.ncbi.nlm.nih.gov/geo/), with accession number GSE193703. The remaining data are available within the Article, Supplementary Information or Source Data file. Source data are provided with this paper.
