# Supplementary material for: KRAS allelic imbalance drives tumour initiation yet suppresses metastasis in colorectal cancer in vivo
Source: Nat Commun. 2024 Jan 2;15:100. doi: 10.1038/s41467-023-44342-4 (PMC10762264; doi:10.1038/s41467-023-44342-4)
Supplement: Supplementary file 1 — Supplementary Information [file 41467_2023_44342_MOESM1_ESM.pdf]

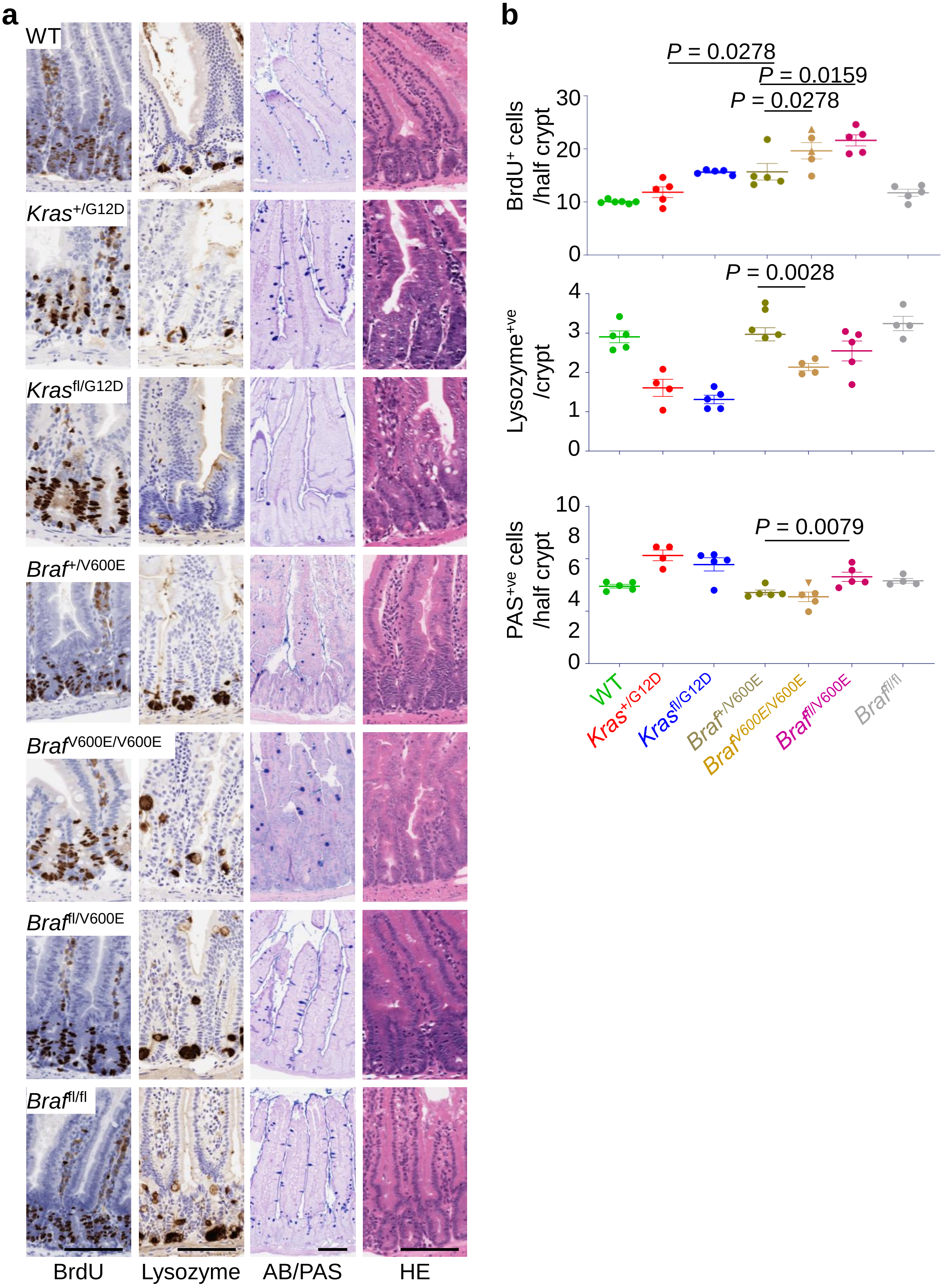
a
WT
Kras+/G12D
Krasfl/G12D
Braf+/V600E
BrafV600E/V600E
Braffl/V600E
Braffl/fl
BrdU
Lysozyme
AB/PAS
HE
b
P = 0.0278
P = 0.0159
P = 0.0278
BrdU+ cells /half crypt
30
20
10
0
P = 0.0028
Lysozyme+ve /crypt
4
3
2
1
0
P = 0.0079
PAS+ve cells /half crypt
10
8
6
4
2
0
WT
Kras+/G12D
Krasfl/G12D
Braf+/V600E
BrafV600E/V600E
Braffl/V600E
Braffl/fl

Supplementary Figure 1: *Braf*^{V600E} gene dosage change alters intestinal homeostasis

a) Representative BrdU, Lysozyme, AB/PAS and H&E IHC of *Kras*$^{+/+}$, *Kras*$^{+/G12D}$, *Kras*$^{fl/G12D}$, *Braf*$^{+/V600E}$, *Braf*$^{V600E/V600E}$, *Braf*$^{fl/V600E}$ and *Braf*$^{fl/fl}$ mouse small intestine, sampled 4 days post Cre-induction. Please note two independent *Braf*^{V600E} alleles were used in this analysis. Scale bar, 100 μm.

b) Top: Quantification of the number of BrdU-positive cells per half-crypt in SI from (a). Data are mean ± s.e.m. *Kras*$^{+/+}$, n = 6, 5M, 1F; *Kras*$^{+/G12D}$, n = 5, 2M, 3F; *Kras*$^{fl/G12D}$, n = 5, 1M, 4F; *Braf*$^{+/V600E}$, n = 5, 1M, 4F; Braf$^{V600E/V600E}$, n = 5, 3M, 2F; *Braf*$^{fl/V600E}$, n = 5, 4M, 1F and *Braf*$^{fl/fl}$, n = 5, 2M, 3F. Middle: Number of Lysozyme-positive cells per half-crypt in SI from (a). Data are mean ± s.e.m. *Kras*$^{+/+}$, n = 5, 5M; *Kras*$^{+/G12D}$, n = 4, 3M, 1F; *Kras*$^{fl/G12D}$, n = 5, 1M, 4F; *Braf*$^{+/V600E}$, n = 5, 1M, 4F; *Braf*$^{V600E/V600E}$, n = 4, 1M, 3F; *Braf*$^{fl/V600E}$, n = 5, 4M, 1F and *Braf*$^{fl/fl}$ n = 4, 3M, 1F. Bottom: Number of AB/PAS-positive cells per half-crypt in SI from (a). Data are mean ± s.e.m. *Kras*$^{+/+}$, n = 5, 5M; *Kras*$^{+/G12D}$, n = 4, 3M, 1F; *Kras*$^{fl/G12D}$, n = 5, 1M, 4F; *Braf*$^{+/V600E}$, n = 5, 3M, 2F; *Braf*$^{V600E/V600E}$, n = 5, 2M, 3F; *Braf*$^{fl/V600E}$, n = 5, 4M, 1F and *Braf*$^{fl/fl}$, n = 4, 3M, 1F. Triangles denote mice induced with 1 mg tamoxifen I.P. All *P*-values generated using one-way Mann–Whitney U test. Source data are provided as a Source Data file.

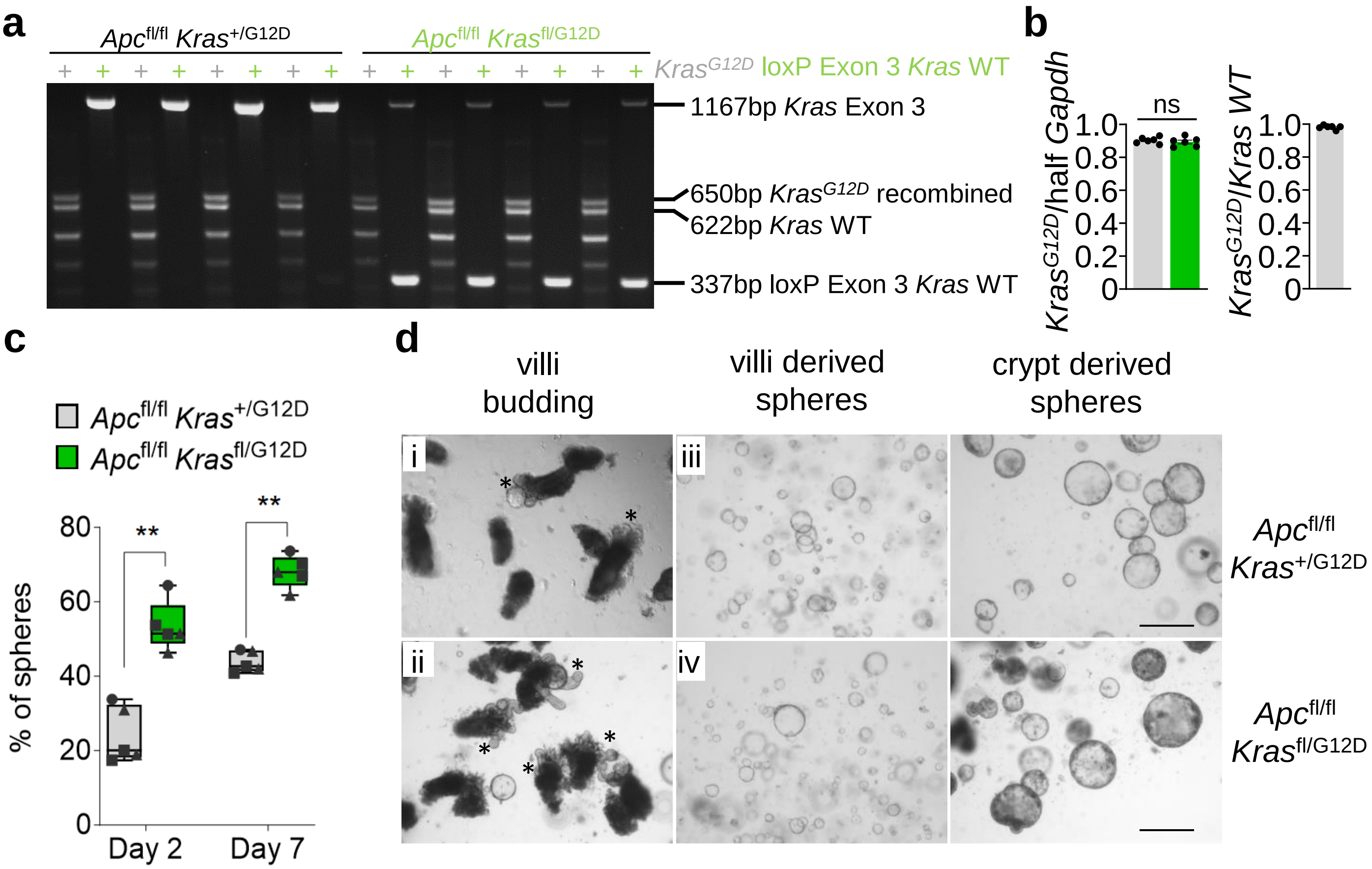

Supplementary Figure 2: Increased de-differentiation capacity of *Apc Kras*^{G12D} villi lacking wild-type *Kras*

a) PCR analysis of *Kras* loci in *Apc*$^{fl/fl}$ *Kras*$^{G12D/+}$ and *Apc*$^{fl/fl}$ *Kras*$^{fl/G12D}$ organoids showing the recombined *Kras*^{G12D} and *Kras*^{fl} (*Kras* exon 3 deletion). Analysis performed on one occasion with 4 independent organoid lines per genotype.

b) Bar graph showing KrasG12D copy number in *Apc*$^{fl/fl}$ *Kras*$^{G12D/+}$ and *Apc*$^{fl/fl}$ *Kras*$^{fl/G12D}$ organoids, expressed relative to half of GAPDH. In most of the cases analyzed, the Kras showed no mutant amplification. *Apc*$^{fl/fl}$ *Kras*$^{+/G12D}$, n = 6, 2M, 4F; *Apc*$^{fl/fl}$ *Kras*$^{fl/G12D}$, n = 6, 2M, 4F. *ns*=not significant, one-way Mann–Whitney U test. Kras-G12D relative to wild-type in *Apc*$^{fl/fl}$ *Kras*$^{G12D/+}$ showing *Kras* allelic balance.

c) Boxplots showing percentage of spheres forming from *Apc*$^{fl/fl}$ *Kras*$^{+/G12D}$ (n=3, 3F) and *Apc*$^{fl/fl}$ *Kras*$^{fl/G12D}$ (n = 3, 1M, 2F) murine intestinal-derived organoids at day 2 and day 7 after seeding. Boxes depict interquartile range, central line indicates median and whiskers indicate minimum/maximum values, representative of 5 technical replicates of organoids generated from 3 individual mice per genotype. ***P* = 0.004 (day2, day7) one-way Mann–Whitney U test.

d) Representative images of *Apc*$^{fl/fl}$ *Kras*$^{+/G12D}$ and *Apc*$^{fl/fl}$ *Kras*$^{fl/G12D}$ villi and crypt derived organoid spheres. Representative of 5 technical replicates. Asterisks marks budding spheres. Source data are provided as a Source Data file.

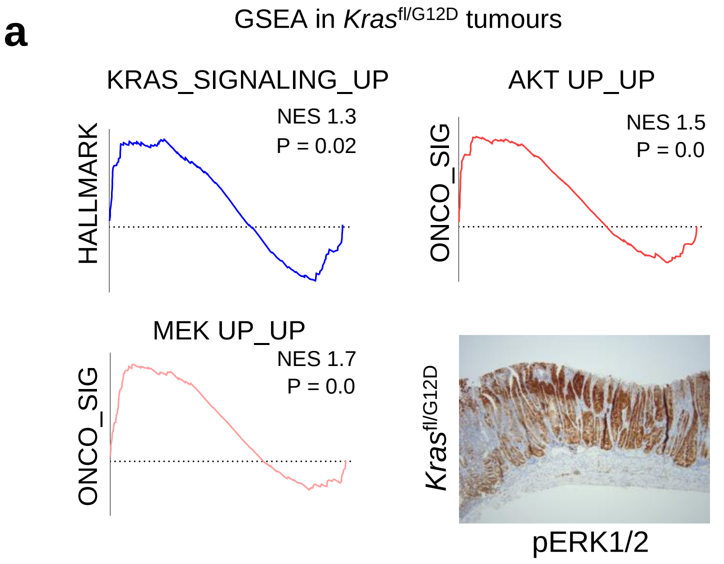

Supplementary Figure 3: Loss of wild-type *Kras* in Kras^{G12D} mutant intestinal tumours leads to transcriptional enrichment of effector pathways.

a) GSEA with the KEGG, Hallmark and oncogenic signatures collection yields an array of highly significant enriched gene sets in *Villin*^{CreER} *Kras*$^{fl/G12D}$ tumours. Selection of enrichment plots representing *KRAS*, *MEK* and *AKT* signalling. *X*-axis shows normalised enrichment score (NES), and the *P* value (computed and corrected for multiple testing using the Benjamini–Hochberg procedure). Source data are provided as a Source Data file.

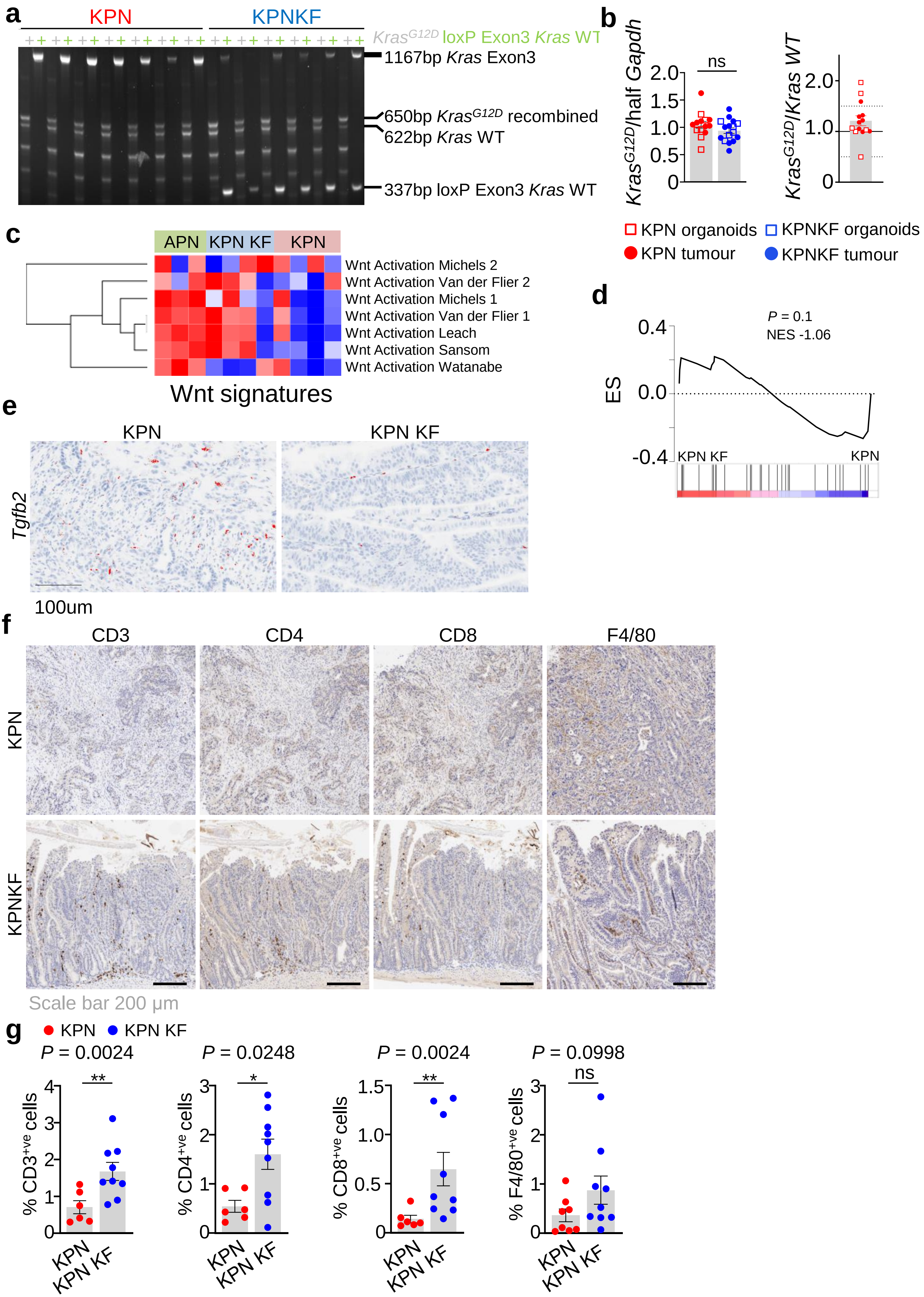

a
KPN
KPNKF
$Kras^{G12D}$ loxP Exon3 Kras WT
1167bp Kras Exon3
650bp $Kras^{G12D}$ recombined
622bp Kras WT
337bp loxP Exon3 Kras WT
b
$Kras^{G12D}$/half Gapdh
ns
$Kras^{G12D}$/Kras WT
KPN organoids
KPNKF organoids
KPN tumour
KPNKF tumour
c
APN
KPN KF
KPN
Wnt Activation Michels 2
Wnt Activation Van der Flier 2
Wnt Activation Michels 1
Wnt Activation Van der Flier 1
Wnt Activation Leach
Wnt Activation Sansom
Wnt Activation Watanabe
Wnt signatures
d
P = 0.1
NES -1.06
ES
KPN KF
KPN
e
KPN
KPN KF
Tgfb2
100um
f
CD3
CD4
CD8
F4/80
KPN
KPNKF
Scale bar 200 μm
g
KPN
KPN KF
P = 0.0024
P = 0.0248
P = 0.0024
P = 0.0998
% $CD3^{+ve}$ cells
% $CD4^{+ve}$ cells
% $CD8^{+ve}$ cells
% $F4/80^{+ve}$ cells
**
*
**
ns

Supplementary Figure 4: Wild-type *Kras* deletion promotes WNT activation and alters TGF*β* levels in KPN tumours

a) PCR analysis of *Kras* loci in KPN and KPN KF organoids showing the recombined *Kras*^{G12D} and *Kras*^{fl} (*Kras* exon 3 deletion). Analysis performed on one occasion with 7 independent KPN and 6 independent KPN KF organoid lines.

b) Bar graph showing KrasG12D copy number in KPN and KPN KF organoids and tumours, expressed relative to half of GAPDH. In most of the cases analyzed, the *Kras* showed no mutant amplification. KPN n = 14 (Tumours, n = 8, 2M, 6F; Organoids, n = 6, 4M, 2F); KPN KF n = 16 (Tumours, n = 10, 4M, 6F; Organoids, n = 6, 3M, 3F), *P*=0.07, one-way Mann–Whitney U test.

c) Heatmap of WNT Activation signatures for *Apc*$^{+/fl}$ *Trp53*$^{fl/fl}$ *Rosa26*$^{N1icd/+}$ (APN), *Kras*$^{fl/G12D}$ *Trp53*$^{fl/fl}$ *Rosa26*$^{N1icd/+}$ (KPN KF) and *Kras*$^{+/G12D}$ *Trp53*$^{fl/fl}$ *Rosa26*$^{N1icd/+}$ (KPN) tumours.

d) GSEA of hallmark *Tgfβ* signalling (GSE15871) in KPN KF tumour organoids compared to KPN. *X*-axis shows normalised enrichment score (NES), and the *P* value (computed and corrected for multiple testing using the Benjamini–Hochberg procedure).

e) Representative *Tgfβ2* ISH in KPN (n = 4, 1M, 3F) and KPN KF (n = 6, 3M, 3F) tumours. Scale bar, 100 µm.

f) Representative staining for CD3, CD4, CD8 and F4/80 IHC in ageing or vehicle treated KPN and KPN KF tumours. Scale bar, 200 µm.

g) Bar graph showing the quantifications of staining from (f). CD3 – KPN, n = 6, 2M, 4F; KPN KF, n = 9, 4M, 5F. CD4 – KPN, n = 6, 2M, 4F; KPN KF, n = 9, 4M, 5F. CD8 – KPN, n = 6, 2M, 4F; KPN KF, n = 9, 4M, 5F. F4/80 – KPN, n = 8, 3M, 5F; KPN KF, n = 9, 4M, 5F. *P*-values calculated via one-way Mann–Whitney U test. Source data are provided as a Source Data file.

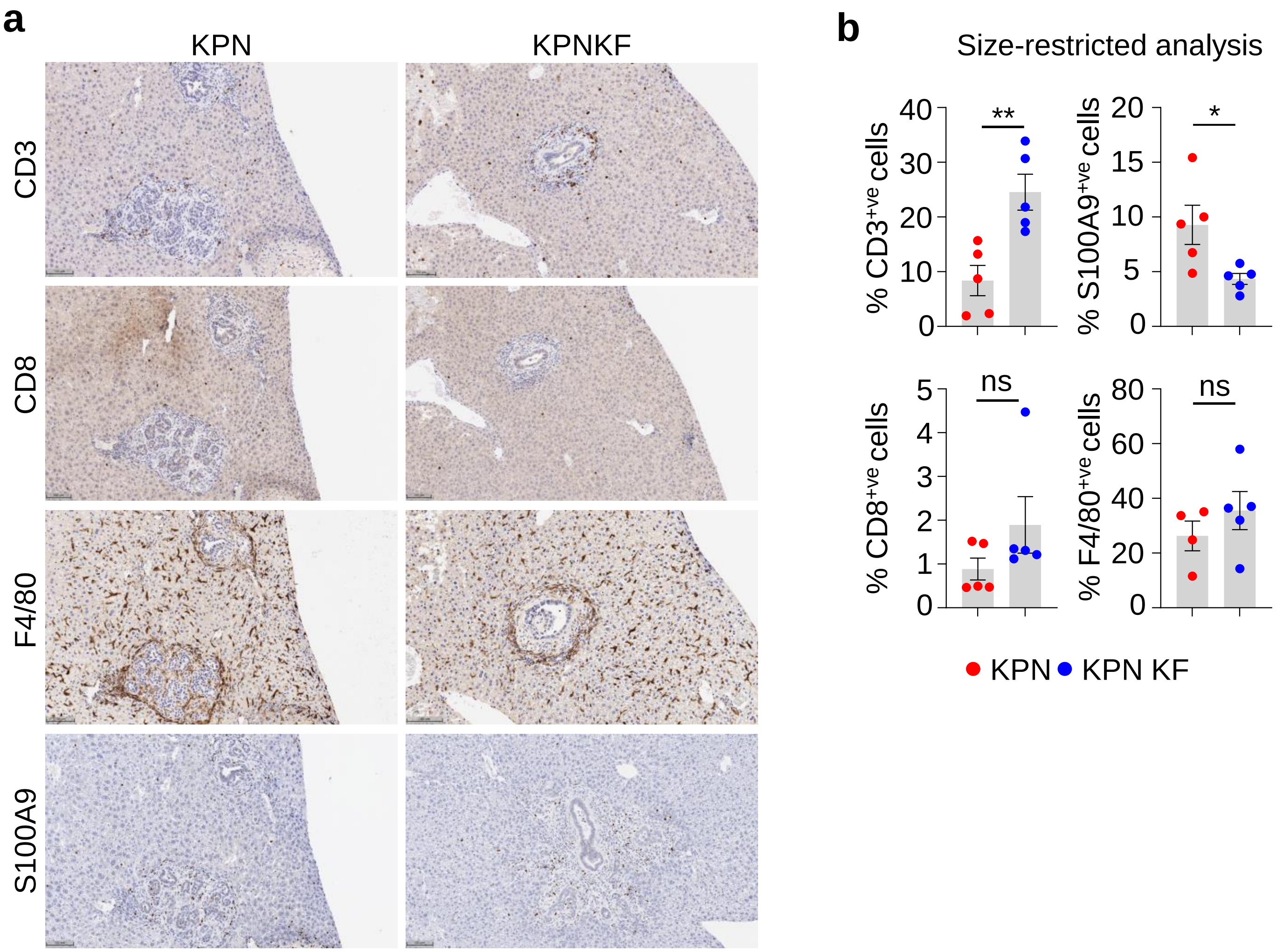

Supplementary Figure 5: Wild-type *Kras* deficient KPN cells show increased immune infiltration also in size matched tumours

a) Representative CD3, CD8, F4/80 and S100A9 IHC following orthotopic transplantation of KPN or KPN KF tumouroids into wild-type recipients. Scale bar, 100 µm.

b) Bar graph showing the quantifications from (**a**) of KPN and KPN KF tumours. Data are mean ± s.e.m. KPN n = 5, 5M, KPN KF n = 5, 5F (CD3, CD8 and S100A8), KPN n = 4, 4M, KPN KF n = 5, 5F (F4/80), $^{**}P = 0.0079$, $^{*}P = 0.0159$, ns = non-significant, one-way Mann–Whitney U test. Source data are provided as a Source Data file.
