# Supplementary material for: KRAS allelic imbalance drives tumour initiation yet suppresses metastasis in colorectal cancer in vivo
Source: Nat Commun. 2024 Jan 2;15:100. doi: 10.1038/s41467-023-44342-4 (PMC10762264; doi:10.1038/s41467-023-44342-4)
Supplement: Supplementary file 2 — Reporting Summary [file 41467_2023_44342_MOESM2_ESM.pdf]

nature portfolio

Corresponding author(s): Arafath K. Najumudeen, Andrew D. Campbell, Owen J. Sansom

Last updated by author(s): 09/11/2023

Reporting Summary

Nature Portfolio wishes to improve the reproducibility of the work that we publish. This form provides structure for consistency and transparency in reporting. For further information on Nature Portfolio policies, see our Editorial Policies and the Editorial Policy Checklist.

Statistics

For all statistical analyses, confirm that the following items are present in the figure legend, table legend, main text, or Methods section.

n/a	Confirmed	
☐	☒	The exact sample size (*n*) for each experimental group/condition, given as a discrete number and unit of measurement
☐	☒	A statement on whether measurements were taken from distinct samples or whether the same sample was measured repeatedly
☐	☒	The statistical test(s) used AND whether they are one- or two-sided<br>*Only common tests should be described solely by name; describe more complex techniques in the Methods section.*
☒	☐	A description of all covariates tested
☐	☒	A description of any assumptions or corrections, such as tests of normality and adjustment for multiple comparisons
☐	☒	A full description of the statistical parameters including central tendency (e.g. means) or other basic estimates (e.g. regression coefficient) AND variation (e.g. standard deviation) or associated estimates of uncertainty (e.g. confidence intervals)
☐	☒	For null hypothesis testing, the test statistic (e.g. *F*, *t*, *r*) with confidence intervals, effect sizes, degrees of freedom and *P* value noted<br>*Give P values as exact values whenever suitable.*
☒	☐	For Bayesian analysis, information on the choice of priors and Markov chain Monte Carlo settings
☒	☐	For hierarchical and complex designs, identification of the appropriate level for tests and full reporting of outcomes
☒	☐	Estimates of effect sizes (e.g. Cohen's *d*, Pearson's *r*), indicating how they were calculated

Our web collection on statistics for biologists contains articles on many of the points above.

Software and code

Policy information about availability of computer code

Data collection	AppliedBiosystems StepOnePlus PCR system using StepOne software version 2.3 (qPCR)
Data analysis	Data analysis was undertaken using Microsoft Excel 2016, Graphpad Prism version 9, ImageScope (v12.3.3.5048), HALO V2.0.1145 (Indica Labs), FastQC v0.11.8, DESeq2 v1.22.2, edgeR v3.28.1, Limma, R v3.6.0

For manuscripts utilizing custom algorithms or software that are central to the research but not yet described in published literature, software must be made available to editors and reviewers. We strongly encourage code deposition in a community repository (e.g. GitHub). See the Nature Portfolio guidelines for submitting code & software for further information.

Data

Policy information about availability of data

All manuscripts must include a data availability statement. This statement should provide the following information, where applicable:

- Accession codes, unique identifiers, or web links for publicly available datasets
- A description of any restrictions on data availability
- For clinical datasets or third party data, please ensure that the statement adheres to our policy

RNA sequencing datasets are archived at GEO (https://www.ncbi.nlm.nih.gov/geo/), with accession number GSE193703. All other data are available as source data accompanying this manuscript, or from the authors on request.

Uncropped versions of blots in Figure 2c, f are provided in the Source Data File.

Research involving human participants, their data, or biological material

Policy information about studies with human participants or human data. See also policy information about sex, gender (identity/presentation), and sexual orientation and race, ethnicity and racism.

Reporting on sex and gender	NA
Reporting on race, ethnicity, or other socially relevant groupings	NA
Population characteristics	NA
Recruitment	NA
Ethics oversight	NA

Note that full information on the approval of the study protocol must also be provided in the manuscript.

Field-specific reporting

Please select the one below that is the best fit for your research. If you are not sure, read the appropriate sections before making your selection.

☒ Life sciences ☐ Behavioural & social sciences ☐ Ecological, evolutionary & environmental sciences

For a reference copy of the document with all sections, see nature.com/documents/nr-reporting-summary-flat.pdf

Life sciences study design

All studies must disclose on these points even when the disclosure is negative.

Sample size	For all in vivo experiments, power analyses were carried out to determine cohort sizes based upon effect size and SD derived from unpublished experiments in similar GA models previously carried out within the lab, and from early pilot studies which were carried out within experimental and control cohorts. Power analyses were carried out using the G* power software package 3.1.9.4 (HHU Dusseldorf), typically defining alpha=0.05 and beta=0.2. Animal studies were also carried out respecting the limited use of animals in line with the 3R system: Replacement, Reduction, Refinement.
Data exclusions	No data were excluded.
Replication	For all in vivo and ex vivo experiments carried out, individual animals of control and experimental cohorts are biologically unique - here replicate data represents analysis of data/samples from independent replicate animals and is denoted by "n".
Randomization	To minimise genetic variability, all experimental and control animals were either generated on a pure, inbred genetic background, or where that was not possible, were generated from individual breeding colonies. Control and experimental animals were co-housed independent of genotype and cohorts were comprised of a balance of both male and female animals. in order to reduce the impact of covariates such as gender or housing, animals were recruited to treatment groups in a partially randomised manner while taking these factors into account.
Blinding	For animal welfare reasons, researchers were not blinded to genotype during study and data collection. The investigator(s) were blinded to genotype or treatment during data analysis.

Reporting for specific materials, systems and methods

We require information from authors about some types of materials, experimental systems and methods used in many studies. Here, indicate whether each material, system or method listed is relevant to your study. If you are not sure if a list item applies to your research, read the appropriate section before selecting a response.

Materials & experimental systems

n/a	Involved in the study
☐	☒ Antibodies
☐	☒ Eukaryotic cell lines
☒	☐ Palaeontology and archaeology
☐	☒ Animals and other organisms
☒	☐ Clinical data
☒	☐ Dual use research of concern
☒	☐ Plants

Methods

n/a	Involved in the study
☒	☐ ChIP-seq
☒	☐ Flow cytometry
☒	☐ MRI-based neuroimaging

Antibodies

Antibodies used

Antibody, catalog number and dilutions used for immunohistochemistry included in the manuscript -
BrdU (BD Biosciences #347580, 1/200 dilution)
Lysosyme (Dako #A0099, 1:500)
pERK (Thr 202/204) (CST #9101, 1:1000)
cMYC (abcam #ab32072, 1:1000)
β-catenin (BD Biosciences #610154, 1:50)
CD3 (Abcam #ab16669, 1:100)
aSMA (Sigma-Aldrich #A2547, 1:100)
CD4 (eBioscience #14-9766-82, 1:100)
CD8a (eBioscience #14-0808-82, 1:100)
F4/80 (Abcam #ab6640, 1:100)
γH2AX (CST #9718, 1:120)
S100A9 (CST #73425, 1:1500)

Antibody, catalog number and dilutions used for immunoblotting -
KrasG12D (CST #14429, 1:1000)
ERK1/2 (CST #4695, 1:1000)
pERK (Thr202/204) (CST#9101, 1:1000)
pMEK1/2 (CST #2338, 1:1000)
MEK1/2 (CST #8727, 1:1000)
pAKT (Ser473) (CST #4060, 1:2000)
AKT (CST #9272, 1:1000)
PTEN (CST #9188, 1:1000)
PAN Ras (Cytoskeleton #AESA02, 1:250)
Anti-Mouse Secondary Antibody (CST #7076s 1:1000)
Anti-Rabbit Secondary Antibody (CST #7074s 1:2000)
Beta-actin (Sigma #A2228)

Validation

Antibodies with catalog number included in the manuscript -
1. BrdU (BD Biosciences #347580); validated by Flow Cytometry. Species Reactivity: Mouse
2. Lysosyme (Dako #A0099); validated by WB, IHC, IP, IF. Species Reactivity: Human, Mouse.
3. pERK (Thr 202/204) (CST #9101); validated by WB, IHC, IF. Species Reactivity: Human, Mouse, Rat, Hamster, Monkey, Mink, Drosophila, Zebrafish, Bovine, Pig and C. elegans.
4. cMYC (abcam #ab32072); validated by Flow Cytometry, WB, IHC, IF. Species Reactivity: Human, Mouse, Rat.
5. β-catenin (BD Biosciences #610154); validated by WB, IHC, IP, IF. Species Reactivity: Human, Mouse, Rat, Dog, Chicken.
6. aSMA (Sigma-Aldrich #A2547); validated by IHC. Species Reactivity: human, mouse, rat, chicken, frog, canine, rabbit, guinea pig, goat, bovine, sheep, snake
7. CD3 (Abcam #ab16669); validated by WB, IHC, IP, IF. Species Reactivity: Human, Mouse, Rat.
8. CD4 (eBioscience #14-9766-82); validated by WB, IHC, IP, IF. Species Reactivity: Human, Mouse, Rat.
9. CD8a (eBioscience #14-0808-82); validated by WB, IHC, IP, IF. Species Reactivity: Human, Mouse, Rat.
10. F4/80 (Abcam #ab6640); validated by Flow Cytometry, IF. Species Reactivity: Mouse.
12. γH2AX (CST #9718); validated by WB, IHC, IF, Flow Cytometry. Species Reactivity Human, Mouse, Rat, Monkey
13. S100A9 (CST #73425); validated by WB, IHC, IF, Flow Cytometry. Species reactivity, Mouse, Rat
14. EnVision Secondary anti-rabbit (DAKO #K4003)
15. EnVision Secondary anti-mouse (DAKO #K4001)
16. KrasG12D (CST #14429); validated by WB. Species reactivity: Human.
17. ERK1/2 (CST #4695); validated by WB, IHC, IP, IF, Flow Cytometry. Species reactivity: Human, Mouse, Rat, Hamster, Monkey, Mink, D.melanogaster, Zebrafish, Bovine, Dog, Pig, C.elegans.
18. pMEK1/2 (CST #2338); validated by WB, IHC, Flow Cytometry. Species reactivity: Human, Mouse, Rat, Monkey.
19. MEK1/2 (CST #8727); validated by WB, IHC, Flow Cytometry. Species reactivity: Human, Mouse, Rat, Monkey, D.melanogaster.
20. pAKT (Ser473) (CST #4060); validated by WB, IHC, IP, IF, Flow Cytometry. Species reactivity: Human, Mouse, Rat, Hamster, Monkey, D.melanogaster, Zebrafish, Bovine.

21. AKT (CST #9272); validated by WB, IP, IF, Flow Cytometry. Species reactivity: Human, Mouse, Rat, Hamster, Monkey, Chicken, D.melanogaster, Bovine, Dog, Pig, Guinea Pig.
22. PTEN (CST #9188); validated by WB, IP, IHC. Species reactivity: Human, Mouse, Rat, Monkey, Dog.
23. PAN-RAS (Cytoskeleton #AESA02); Information not available.
24. Beta-actin (Sigma #A2228); validated by WB, IP, IHC, IF. Species reactivity: Human, Bovine, Sheep, Pig, Rabbit, Cat, Dog, Mouse, Rat, Guinea Pig, Chicken, Carp, Leech.
25. Anti-Mouse Secondary Antibody (CST #7076); validated by WB. Species reactivity: Mouse.
26. Anti-Rabbit Secondary Antibody (CST #7074); validated by WB. Species reactivity: Rabbit.

Eukaryotic cell lines

Policy information about cell lines and Sex and Gender in Research

Cell line source(s)	3D organoid lines from mouse intestinal crypts were derived at the Cancer Research UK Scotland Institute
Authentication	Organoid lines were authenticated by PCR genotyping
Mycoplasma contamination	All cell lines are routinely tested for mycoplasma and it is considered that the lines used in the study are mycoplasma free.
Commonly misidentified lines (See ICLAC register)	No commonly misidentified lines were used in the study.

Animals and other research organisms

Policy information about studies involving animals; ARRIVE guidelines recommended for reporting animal research, and Sex and Gender in Research

Laboratory animals	Male and female mice from 6 to 15 weeks old and of defined genetic background were used, these are described in methods and figure legends. For intestinal studies, the transgenes and conditional alleles used were as follows – villin-creERT2, Apc-fl, Kras-LSL-G12D, Braf-V600E(tm1Mmcm), Braf-V600E(tm1Cpri), Trp53-fl and Rosa26-N1icd. Mice carrying these were interbred as described to generate all experimental lines used in this study.
Wild animals	No wild animals were used in the study
Reporting on sex	Both male and female mice were used for all in vivo and ex vivo experiments.
Field-collected samples	No field-collected samples were used in this study.
Ethics oversight	All experiments were performed according to UK Home Office regulations (Project licences 70/8646, PP3908577), under the oversight of the Animal Welfare and Ethical Review Board (AWERB) of the University of Glasgow.

Note that full information on the approval of the study protocol must also be provided in the manuscript.

Plants

Seed stocks	*Report on the source of all seed stocks or other plant material used. If applicable, state the seed stock centre and catalogue number. If plant specimens were collected from the field, describe the collection location, date and sampling procedures.*
Novel plant genotypes	*Describe the methods by which all novel plant genotypes were produced. This includes those generated by transgenic approaches, gene editing, chemical/radiation-based mutagenesis and hybridization. For transgenic lines, describe the transformation method, the number of independent lines analyzed and the generation upon which experiments were performed. For gene-edited lines, describe the editor used, the endogenous sequence targeted for editing, the targeting guide RNA sequence (if applicable) and how the editor was applied.*
Authentication	*Describe any authentication procedures for each seed stock used or novel genotype generated. Describe any experiments used to assess the effect of a mutation and, where applicable, how potential secondary effects (e.g. second site T-DNA insertions, mosiacism, off-target gene editing) were examined.*
